# Portable sequencing as a teaching tool in conservation and biodiversity research

**DOI:** 10.1371/journal.pbio.3000667

**Published:** 2020-04-16

**Authors:** Mrinalini Watsa, Gideon A. Erkenswick, Aaron Pomerantz, Stefan Prost

**Affiliations:** 1 Field Projects International, Saint Louis, Missouri, United States of America; 2 Population Sustainability, San Diego Zoo Global, San Diego, California, United States of America; 3 Department of Biology, University of Missouri–St. Louis, St. Louis, Missouri, United States of America; 4 Infectious Diseases, School of Medicine, Washington University in St. Louis, St. Louis, Missouri, United States of America; 5 Department of Integrative Biology, University of California, Berkeley, Berkeley, California, United States of America; 6 Marine Biological Laboratory, Woods Hole, Massachusetts, United States of America; 7 LOEWE-Centre for Translational Biodiversity Genomics, Senckenberg, Frankfurt, Germany; 8 South African National Biodiversity Institute, National Zoological Garden, Pretoria,South Africa

## Abstract

As biodiversity loss continues to accelerate, there is a critical need for education and biomonitoring across the globe. Portable technologies allow for in situ molecular biodiversity monitoring that has been historically out of reach for many researchers in habitat nations. In the realm of education, portable tools such as DNA sequencers facilitate in situ hands-on training in real-time sequencing and interpretation techniques. Here, we provide step-by-step protocols as a blueprint for a terrestrial conservation genetics field training program that uses low-cost, portable devices to conduct genomics-based training directly in biodiverse habitat countries.

## Abstract 2

As biodiversity loss continues to accelerate, there is a critical need for education and biomonitoring across the globe. This Community Page article presents step-by-step protocols as a blueprint for a terrestrial conservation genetics training program that uses low-cost, portable devices to conduct genomics-based training directly in the field.

## Introduction

We live in an era characterized by major environmental change, large-scale defaunation, and biodiversity loss [1,2]. The immense task of quantifying the status quo of global ecosystems and the rate of biodiversity loss has been reinforced by developments in high-throughput DNA sequencing (HTS) technologies, which are increasingly being utilized as tools for wildlife conservation [3]. However, researchers and science educators in remote areas can find it difficult to access HTS, often due to a combination of high costs, bulky equipment, and lack of infrastructure, resulting in a high level of dependency on foreign service providers [4,5].

Recent technological innovations, such as portable, low-cost instruments enabling next-generation sequencing in remote environments, are transforming this landscape [6]. A critical advancement is the development of Oxford Nanopore Technologies’ (ONT’s) MinION sequencing platform, which has many advantages compared to other HTS devices. First, the sequencer and 2 flow cells start at an initial cost of US$1,000 and allow for multiplexing of samples within flowcells (generating a maximum output of approximately 10–20 Gigabases [Gb]), further reducing per sample costs. Today, a cheaper flowcell (the Flongle) with a reported output of approximately 1–2 Gb is available, which can be ideal for smaller educational DNA barcoding or metabarcoding projects. Second, the approximately 90-g MinION is powered through a laptop’s USB port, making it both energy efficient and portable. However, its greatest advantage to conservationists is the possibility of conducting in situ sequencing. It has been deployed successfully in a range of field conditions, including real-time sequencing of endangered wildlife in the forests of Ecuador [7], Madagascar [8], and Tanzania [9]. Supporting devices have also become low-cost, portable, and rugged. PCR machines (e.g., MiniPCR, MiniOne; range US$650–US$900) that can be controlled by smartphones and run from simple battery packs, have been field-tested [7]. Small desktop centrifuges, with either a fixed or variable speed, are available from multiple vendors (cost US$150–US$350), but it is even possible to 3D print a manually powered do-it-yourself centrifuge (e.g., the paperfuge [10]). All costs listed were sourced at the time of publication. Thus, the MinION and associated portable equipment can help to minimize sample storage times, sample transport, and associated costs and eliminate the need to export biological specimens across country borders. It must be noted that permits to access and use genetic resources from biological specimens might still be required, as per the Nagoya Protocol of the Convention on Biological Diversity and local and national regulations.

**Fig 1:**
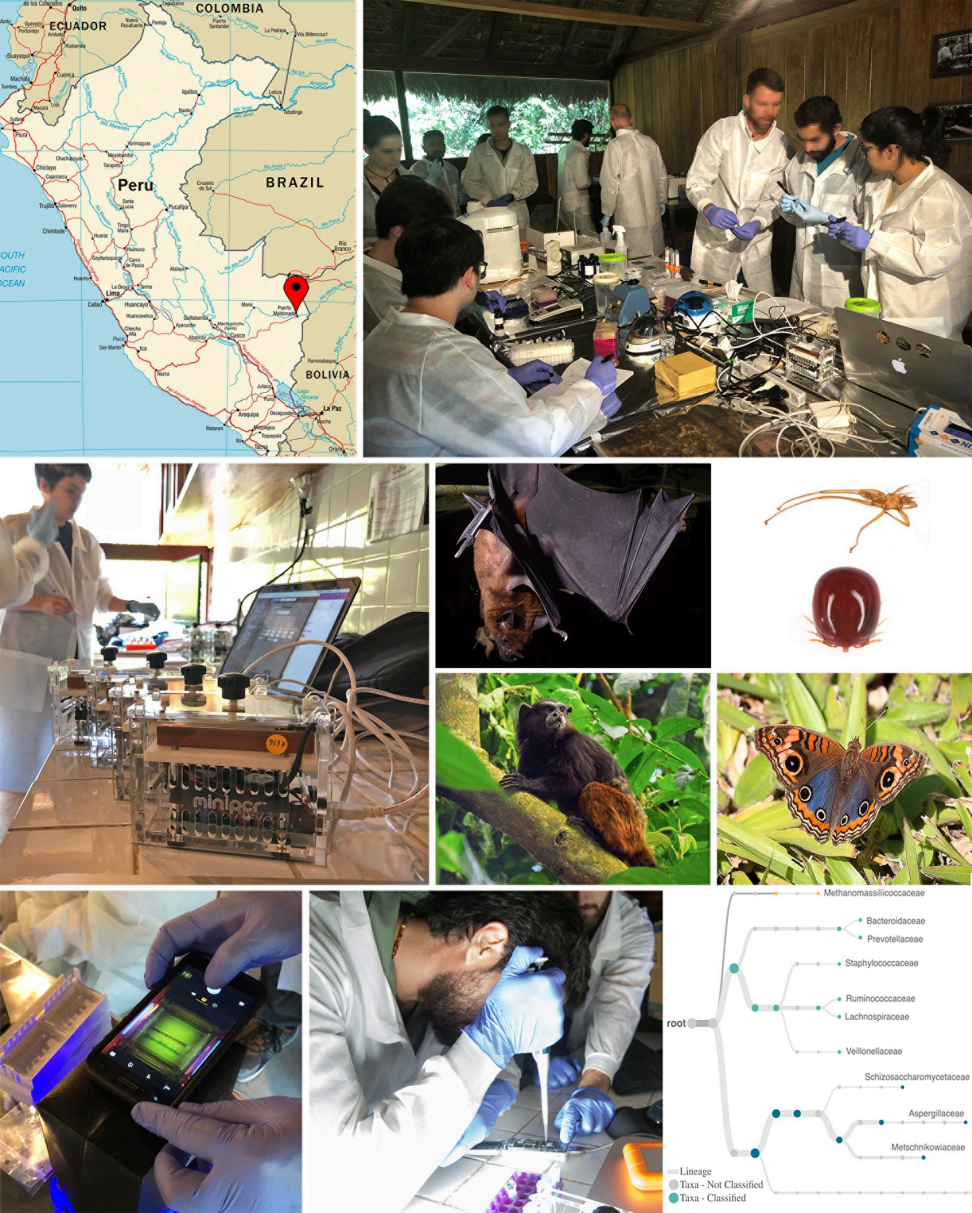
Biomonitoring training programs and examples from case studies. Top: the location and setup of the Green Lab, a molecular genomics field laboratory in southeastern Peru. Middle: portable PCR devices analyzing locally collected specimens, including a bat and its ectoparasite, a saddleback tamarin primate, and a butterfly, which were photographed in the Madre de Dios region of Peru. Bottom: imaging of amplicons during agarose gel electrophoresis using smartphones, loading of a flowcell with a field-prepped library onto ONT’s MinION portable sequencer, and the output from WIMP [13] of the phylogenetic analysis of the fecal microbiome analyzed during a field training program conducted at the Green Lab. Data for Fig 1 are provided in S1 Data. ONT, Oxford Nanopore Technologies; WIMP, What’s in my Pot?. *Image Credit*: *Bat*: *Ishaan Raghunandan; Tamarin*: *Timothy Paine; Arthropod ectoparasites*, *butterfly*, *and laboratory images*: *Aaron Pomerantz*.

Portable technologies have also been utilized as effective and affordable teaching tools, but publications recapping lesson plans have mostly been from classroom or laboratory settings [11,12]. Here, we outline a blueprint for a conservation genetics field training program based on previous experience conducting molecular biodiversity research in remote areas and 2 field training programs conducted at a field laboratory in the Peruvian Amazon rainforest (2018–2019) (Fig 1). By publishing the program plan and outcomes, we invite a discussion on the practicality, as well as the costs and benefits, of providing training in applied field genomics at remote sites or in countries with limited access to sequencing facilities to participants of varying backgrounds.

## Why implement in situ training programs?

Short field programs, in which participants travel to natural environments to train, can guide career paths, provide local capacity building, and highlight important environmental problems [14–17]. Bringing the science to the sample, effectively turning the paradigm on its head, also welcomes local scientists and educators who perform invaluable environmental monitoring without contemporary methodologies or large research funding. Critically, these programs can attract a wide range of national and international participants from academic, commercial, or private backgrounds.

**Fig 2:**
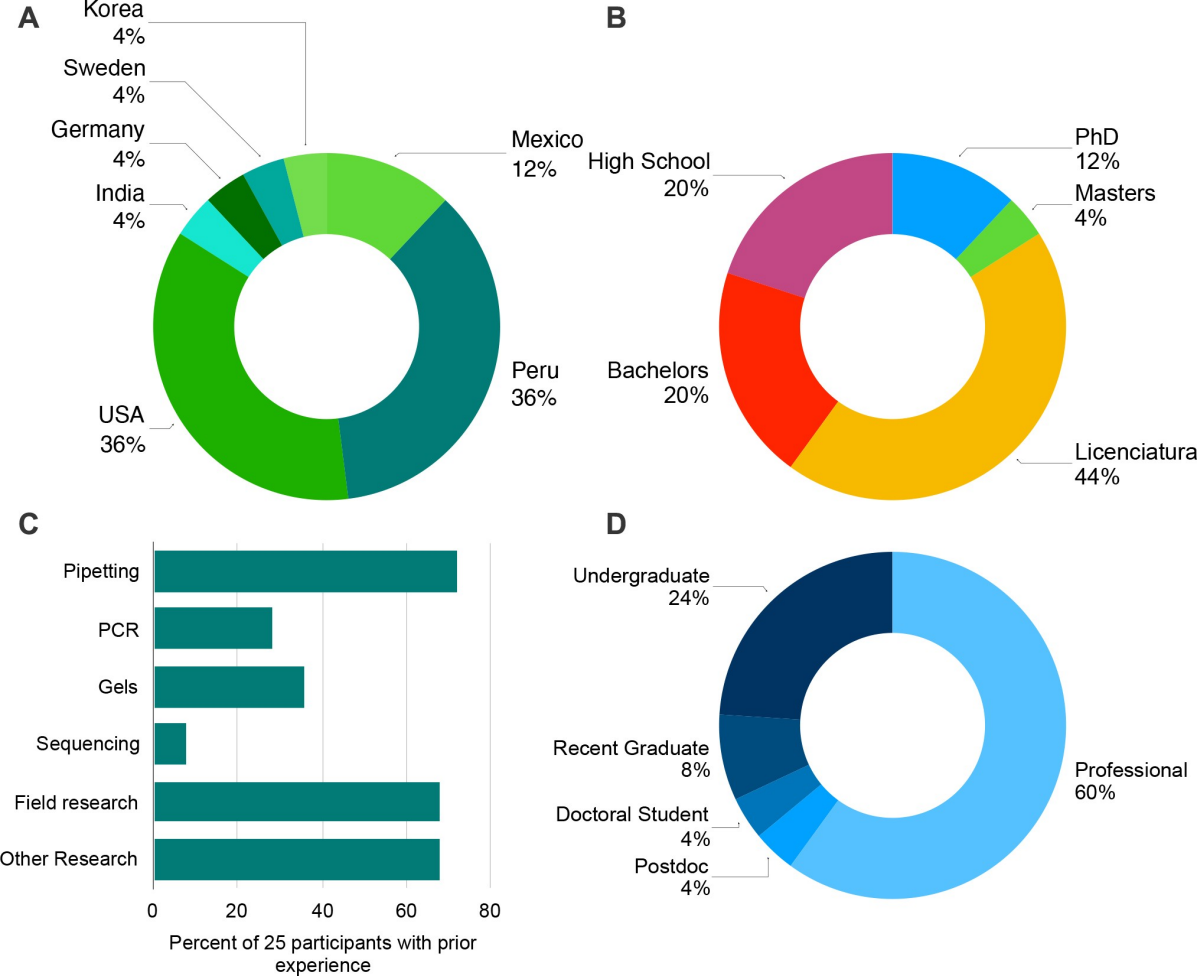
Demographics of attendees of conservation genomics training programs (*n* = 25) hosted at the Green Lab in southeastern Peru in 2018 and 2019. (A) Participants by nationality. (B) Participants by the highest degree received. Note: a *licenciatura* is more advanced than a Bachelor’s degree in Peru and possibly a Master’s thesis equivalent. (C) Prior laboratory experience of participants. (D) Occupation of each participant at the time of attending the training program.

Our Peruvian training programs were attended by an international audience from 7 countries, with 72% of attendees split equally between Peru and the United States. We did not screen participants based on background or nationality, requiring only that they be over 18 years of age. For every 4 paying participants, we offered a scholarship to one person, first targeting participants from the host country and then more broadly through the Americas. Of the 25 participants, the majority (60%) were professionals in their fields, while the rest were active students (Fig 2). Forty percent identified as female, and the rest identified as male. Conservation scientists, professors of biology, bioinformaticians, and restoration specialists have attended these programs. Sixteen percent of attendees had advanced degrees (PhD or Master’s), while the bulk (40%) had a *licenciatura*, a Peruvian Bachelor’s degree with honors including the completion of an intensive research project postgraduation. Over 60% had some field or laboratory research experience, only approximately 20% had ever run a PCR or gel before, and even fewer had ever sequenced DNA. This diversity in education, experience, occupation, and country of origin allowed us to tailor the program to a wide variety of individuals, from novices to professionals in the field of genetics.

From an instructional perspective, we think that participant diversity is a strong asset to the training program as a whole; indeed, we received only positive feedback from participants with advanced laboratory or bioinformatic experience. All participants gave feedback at the end of their final quiz. A postcourse questionnaire, asking their opinion on how much of a challenge course activities posed, how involved and knowledgeable course instructors were, overall satisfaction, impact on future goals, and whether they would recommend the course to others, was also answered by 10 of the 15 participants in the first program (see S1 Appendix for the anonymized questionnaire and detailed responses). Highly positive feedback was received on project-based learning with real data, use of portable sequencing, the balance between field excursions and laboratory work, the acquisition of new skills, and learning about applications of these skills to conservation genetics test cases. Participants mentioned areas for improvement, including an overwhelming new genetics vocabulary, laboratory work being more intensive than expected, laboratory overcrowding being a stressful factor, and wanting more time to devote to bioinformatics. Based on that, we were able to improve the sharing of samples and laboratory equipment, reduce bench time with fewer case studies, send information on software and basic coding in advance, and reserve 3 days solely for bioinformatics practice in the second course. Since new terminology appeared to be a challenge, we recommend providing a glossary of terms before program inception. To in-country and foreign participants alike, the impact of learning conservation genomics was amplified by being in a habitat of high conservation concern. Using samples from regional wildlife to answer scientific questions of value to local conservationists also makes these field programs meaningful and can result in discoveries publishable by program participants. Participant networks can also produce new research collaborations between individuals and organizations that would not normally form.

Where formalized options for habitat–country research scientists have been lacking, we can now implement hands-on training in real-time DNA sequencing techniques for genomics-based biomonitoring, simultaneously generating a widespread network of laboratory-trained conservation scientists within regions of high biodiversity.

## Where can field genomics programs be implemented?

A formal laboratory space is not a prerequisite for these programs so long as certain basic requirements are fulfilled, including access to the following: (a) reliable power, (b) sufficient bench space, and (c) cold storage. Commodities like an oven/autoclave for equipment sterilization can further reduce long-term costs; alternatively, presterilized materials can be purchased (see S2 Appendix for equipment recommendations). Our programs were implemented in a more formalized laboratory space; in 2018, a still-ongoing collaborative effort between a Peruvian ecotourism company (Inkaterra Hotels) and conservation research organization based in the US (Field Projects International [FPI]) resulted in the installation of the Amazon rainforest's first dedicated field molecular laboratory, called the “Green Lab.” Located an hour's boat ride from the nearest town of Puerto Maldonado in Peru (12° 31′ S, 69° 2' W), the laboratory itself is housed in a two-room wooden cabin with a thatched roof, relatively open to the elements. We found that the close proximity to a larger city helped tremendously with logistics while, at the same time, the location was remote enough to provide the participants with a true rainforest experience. Energy requirements were met by a combination of solar panels and a generator-based power supply, with the intention of switching entirely to solar energy in the near future (see [18] for a list of laboratory capabilities). Although we focus on terrestrial systems, most of the setups and methods can easily be adjusted to studying marine or aquatic biodiversity.

Amenities such as the internet, even if a low-bandwidth service, while not strictly necessary, do simplify instruction and, for example, could be used for troubleshooting tool installations for bioinformatics practicals that arose due to the range of operating systems on participant laptops. To reduce dependency on internet access, we recommend downloading databases for applications like DNA barcoding (see Table 1), such as the Barcode of Life Data System (BOLD) or the National Center for Biotechnology Information (NCBI)’s *nt* database, on local hard drives prior to the program. Furthermore, setting up virtual Linux environments on USB drives can help to overcome issues with installations of bioinformatic tools.

**Table 1:** Overview of common genetic approaches used in biodiversity research and their advantages and disadvantages for field-based education.

Technique	Description of technique	Applications	Advantages	Drawbacks
DNA barcoding	Amplification of a short target region of DNA that contains species diagnostic sites.	(1) Species identification, (2) biodiversity monitoring, and (3) diet and pathogen detection from scat or plants	Easy, fast, and reliable; samples can be multiplexed on a single flowcell	Requires good reference databases.
Metabarcoding	Amplification of DNA barcodes using universal primers to detect many taxa within a bulk community or pooled taxon sample.	(1) Biodiversity monitoring, (2) microbiome analyses, and (3) diet and pathogen detection from scat or plants	Easy, fast, and reliable; samples can be multiplexed on a single flowcell	Requires good reference databases; given that analyses are usually based on individual reads, as opposed to DNA barcoding, the current read error rate might hinder correct species assignment; only one nanopore-specific pipeline available currently (WIMP).
Metagenomics	Shotgun sequencing of total DNA in a bulk community or pooled taxon sample.	(1) Biodiversity monitoring and (2) diet and pathogen detection from scat, tissue, or plants	Easy and fast; samples can be multiplexed on a single flowcell	A current lack of good reference databases; some taxa sequence better than others, which can lead to a skewed representation; high data requirements compared to DNA barcoding or metabarcoding; given that analyses are usually based on individual reads, the current read error rate might hinder correct species assignment.
eDNA	Metabarcoding for environmental samples to pick up trace DNA left by organisms living in the environment.	(1) Biodiversity community monitoring from environmental sources, i.e., water or soil and (2) invasive- or target-species detection in environmental samples	Easy and fast; samples can be multiplexed on a single flowcell	The current read error rate might hinder correct species assignment; no nanopore-specific pipelines available currently.
Genome skimming	Retaining only multicopy loci, such as chloroplast or mitochondrial genomes from metagenomics data.	(1) Species identification, (2) biodiversity monitoring, and (3) diet and pathogen detection from scat or plants	Easy and fast; samples can be multiplexed on a single flowcell	The current lack of reference databases; some taxa sequence better than others, which can lead to a skewed representation; more sequencing data required compared to DNA barcoding or metabarcoding.
Genome sequencing	Sequencing of the entire genome of an organism.	(1) Genome assembly and annotation	Requires only ONT library prep, so it is easy to execute	Requires a more-sophisticated high–molecular weight DNA isolation protocols; typically requires a high amount of sequencing coverage, more data output, and bioinformatics methods.

**Abbreviations:** eDNA, environmental DNA; ONT, Oxford Nanopore Technologies; WIMP, What’s in my Pot?

## Suggested training program outline

We have put together an example program syllabus that can easily be adapted to suit individual course needs (S3 Appendix). We recommend that programs begin with immersion in the local habitat, including excursions and lectures emphasizing local biodiversity and threats. Appreciation for one’s surroundings highlights the uniqueness of the learning opportunity. Next, we suggest reviewing the fundamentals of genetics, including laboratory skills like pipetting or loading gels, and bioinformatics basics. Often, the bioinformatics approach helps participants understand the choice of molecular methods. As the program progresses, we found it helpful to set aside unstructured times that participants could use to digest material or gain one-on-one time with an instructor; the instructor:student ratios were approximately 1:4. We suggest preparing backups for each step in advance to ensure that mistakes and equipment failures during the program do not hinder workflow completion. Sequencing should preferentially occur at least 3 days before program termination for sufficient time to teach bioinformatic analyses and interpret program data. Experimental case studies can either be conducted in small groups (allowing for more case studies and hands-on experience) or large groups (fewer case studies but a more homogenous experience). We conducted training programs with both approaches. However, a suitable model depends on the desired program outcomes and factors such as group size, sample numbers, and prior participant laboratory experience. We chose a 2-week program length based on instructor and participant availability as well as field accommodation costs. We found shorter durations were more conducive to diverse audience participation (e.g., professionals without university breaks) and to lower program fees.

Overall, we designed the program activities to include 15% field work, 20% lectures, 15% bioinformatics, and 50% laboratory work on independent case studies. Participants utilized a mixture of hand-written laboratory notebooks and software programs to keep notes and took 2 quizzes to help instructors evaluate progress and understanding. Evening lectures and daytime laboratory activities were effective for us but should be modified to suit the needs of individual programs.

## Examples of case studies

A variety of sequencing-based experiments exist for conservation education; common methods are summarized in Table 1 with detailed protocols available in S4 Appendix. In our programs, we implemented up to 4 sequencing experiments per program, designed to tackle different conservation genomics challenges and to demonstrate the viability of conducting such analyses in the field. While providing a good overview of techniques in biodiversity research and conservation, fewer case studies allows for deeper immersion into a topic. The most successful projects we found were also those with guaranteed outputs and relatively straightforward bioinformatics pipelines. The most popular of these is DNA barcoding, involving multiplexed amplicon sequencing. It is preferable as a teaching example over others due to its simplicity, well-established protocols, and utility (see Table 1). Participants begin with DNA extraction from various locally collected tissue samples. Simple PCRs for common markers such as *Cytochrome c oxidase subunit I* (animals) or *matk* (plants) can be evaluated via agarose gel electrophoresis. Detectable amplicons are then PCR barcoded using custom or kit-based barcodes (e.g., ONT’s 12-barcode kit or [13]), purified using bead cleanups, and quantified on a device such as a Quantus fluorometer (Promega). After normalization, samples are pooled into one or more libraries and end-prepped and dA-tailed (New England BioLabs) prior to sequencing. Sequencing itself can proceed quickly and can be terminated within a few hours, since 10× to 100× coverage has been shown to provide adequate data for building consensus sequences [19]. In some cases, flowcells can be washed and reused within a few days with different barcodes to prevent cross-run sequence contamination. The development of many bioinformatic pipelines in recent years adds to the utility of DNA barcoding as a case study (see [6] for a review). Off-the-shelf software such as Geneious, UGene, or CLCGenomics are Graphical User Interface (GUI)-based but often pricier alternatives.

Other case studies include 16S metabarcoding of intestinal microbiota to assess gut health and diet or environmental DNA (eDNA) samples such as water and soil. Given the higher error rate of the MinION sequencer, scientific conclusions drawn from the data have to be treated carefully [6,20]. Nevertheless, we feel that these are valuable options for teaching. We also tested more complex protocols such as double-digest restriction site–associated DNA sequencing (ddRAD) using MinION sequencing, but we do not recommend these for first-time biologists taking a more introductory-level genomics training. In addition to more ingredients requiring constant cooling or freezing, and increased program costs, the protocol was difficult to implement and required more sequencing power to produce effective results than was feasible during a short training program. All tested protocols are available for use in a collection on protocols.io: dx.doi.org/10.17504/protocols.io.9dnh25e.

## Conclusion

Portable sequencing technology can help democratize scientific research and conservation efforts. A necessary step to implementing a new conservation tool is training local science educators and conservationists in areas in which research funding and infrastructure are lacking. Here, we have outlined protocols and lessons learned from running a conservation genetics field training program that utilizes low-cost, portable devices to conduct genomics-based training directly in a biodiverse tropical rainforest. We hope that this serves as a resource for others to establish in situ genomics as a teaching tool in both terrestrial and marine conservation and biodiversity research.

## Supporting information

S1 Appendix Postcourse questionnaire and summary statistics. (PDF)

S2 Appendix Equipment lists (requirements and alternatives). (DOCX)

S3 Appendix Generalized course syllabus. (DOCX)

S4 Appendix Protocols, bioinformatic pipelines, and resources for executing case studies during training programs. (PDF)

S1 Data Raw data for Fig 1. (XLSX)

## Abbreviations

BOLD: Barcode of Life Data System
ddRAD: double-digest restriction site–associated DNA sequencing
eDNA: environmental DNA
FPI: Field Projects International
Gb: Gigabases
GUI: Graphical User Interface
HTS: high-throughput DNA sequencing
NCBI: National Center for Biotechnology Information
ONT: Oxford Nanopore Technologies
WIMP: What's in my Pot?
