# Supplementary material for: Portable sequencing as a teaching tool in conservation and biodiversity research
Source: PLoS Biol. 2020 Apr 16;18(4):e3000667. doi: 10.1371/journal.pbio.3000667 (PMC7188297; doi:10.1371/journal.pbio.3000667)
Supplement: S1 Appendix — (PDF) [file pbio.3000667.s001.pdf]

Form Preview : ID 60

☐ display grid ☐ show structure

Note: This is a simple form preview. This form may display differently when added to your page based on normal inheritance from parent theme styles.

Course Evaluation: (Genomics)

Please give us your opinion of the course in the form below. We loved having you participate in the course, and would love to know how it could be improved for future generations of students. Thank you!

Please enter your course name below *

Please enter your course month and year below *

Eg: January, 2018

Section 1 of 4: Do you agree with the following statements?

The readings helped me learn the course material *

- ○ Strongly disagree
- ○ Disagree
- ○ Neutral
- ○ Agree
- ○ Strongly Agree
- ○ Not Applicable

The lectures helped me learn the course material *

- ○ Strongly disagree
- ○ Disagree
- ○ Neutral

Viewport (Width : 1738px , Height :717px)

Agree

Strongly Agree

The material covered in the course was challenging to me *

Strongly disagree

Disagree

Neutral

Agree

Strongly Agree

The course activities were challenging to me *

Strongly disagree

Disagree

Neutral

Agree

Strongly Agree

I received enough attention from the instructor(s) *

Strongly disagree

Disagree

Neutral

Agree

Strongly Agree

The course instructor(s) were sufficiently knowledgeable about the topics taught *

Strongly disagree

Disagree

Neutral

Agree

Viewport (Width : 1738px , Height :717px)

◯ Strongly Agree

Overall, I am satisfied at the end of this course in terms of my expectations when I signed up for it *

◯ Strongly disagree

◯ Disagree

◯ Neutral

◯ Agree

◯ Strongly Agree

This course has helped me figure out more about my interests and possible future goals *

◯ Strongly disagree

◯ Disagree

◯ Neutral

◯ Agree

◯ Strongly Agree

I would recommend this course to someone else *

◯ Strongly disagree

◯ Disagree

◯ Neutral

◯ Agree

◯ Strongly Agree

Section 2 of 4: For the following questions, please select ALL that apply:

When would be the right time to take this course? *

☐ High school graduate (age 17)

☐ Freshman in College (Year 1, age 18)

Viewport (Width : 1738px , Height :717px)

- [ ] Sophomore in College (Year 2, age 19)
- [ ] Junior in College (Year 3, age 20)
- [ ] Senior in College (Year 4, age 21)
- [ ] College graduate (age 22+)
- [ ] Graduate student
- [ ] Professional Advancement
- [ ] No Opinion

Please pick as many of these words that apply to the experience you had on this course: *

- [ ] Amazing
- [ ] Regret
- [ ] Useless
- [ ] Fun
- [ ] Tough
- [ ] Satisfied
- [ ] Fascinating
- [ ] Difficult
- [ ] Unforgettable
- [ ] Boring
- [ ] Useful
- [ ] Forgettable
- [ ] Valuable
- [ ] Tedious
- [ ] Dissatisfied

Please check if your instructor(s) did any of the following: *

- [ ] discussed the FPI sexual harassment policy with participants as a group
- [ ] discussed the FPI sexual harassment policy with you individua

Viewport (Width : 1738px , Height :717px)

- [ ] discussed field safety protocols with the group
- [ ] discussed wildlife handling ethics with the group
- [ ] listened and reacted to concerns you brought up regarding course content
- [ ] listened and reacted to concerns you brought up regarding course social dynamics
- [ ] behaved ethically regarding wildlife handling on the course
- [ ] None of the above

Check as many as apply

Section 3 of 4: Please respond to the following questions, and feel free to be as concise or elaborate as you like.

How would you rate your level of experience with molecular biology techniques before taking this course?

What challenges (if any) did you encounter with yourself, or in the group, while performing experiments in the lab?

In your opinion, how clear were the scientific objectives and methods? If you were to do this all over again, what might you change to make things more clear or straightforward?

Viewport (Width : 1738px , Height :717px)

Now that you have completed the two week "Genomics in the Jungle" course, do you feel more proficient with any of the topics or experiments that you completed? If so, which ones, and which areas do you still wish to work on further?

Real-time portable DNA sequencing can have many applications. What would you consider to be the top 3 most important implementations with portable scientific instruments? Are there any tools that you think could be made more cost-effective / portable in the future?

Section 4 of 4: Please comment on the following and let us know how we did, and if we can improve. Your opinion is very important to us.

The facilities at each of the field sites used on this course. *

Please list at least 2 (more are welcome) specific course activities/events/experiences you enjoyed the MOST and the LEAST. *

Eg: Most enjoyable:.... Least enjoyable:....

Viewport (Width : 1738px , Height :717px)

Please share any specific changes/modifications/additions/eliminations to improve the course itinerary/schedule/activities/exercises. *

Please indicate both POSITIVE and NEGATIVE qualities of the course instructor(s) that could be retained/improved on in future courses. *

Please list the most useful/interesting skills or knowledge you gained during the course *

The cost effectiveness of the course (in comparison to other similar opportunities) *

Please note your most vivid memories from the course (positive or negative) *

Any other comments?

Viewport (Width : 1738px , Height :717px)

Specifically, were there other words you would use to describe your experience on this course?

Would you be interested in contributing a short article for the FPI blog on any of your experiences while attending this course? *

- ◯ Yes
- ◯ No
- ◯ Not sure, tell me more

Submit

Viewport (Width : 1738px , Height :717px)

Appendix D. Table 1: Minimum, maximum and mode values for section 1 of the PCQ evaluated on a 5-point scale: Strongly Disagree, Disagree, Neutral, Agree, and Strongly Agree. PCQ was taken by 10 of the 15 participants in program 1 (2018).

**Question**	**Mode**	**Minimum**	**Maximum**
The readings helped me learn the course material	Agree	Agree	Strongly Agree
The lectures helped me learn the course material	Strongly Agree	Agree	Strongly Agree
The material covered in the course was challenging to me	Agree	Neutral	Strongly Agree
The course activities were challenging to me	Neutral	Neutral	Strongly Agree
I received enough attention from the instructor(s)	Strongly Agree	Neutral	Strongly Agree
The course instructor(s) were sufficiently knowledgeable about the topics taught	Strongly Agree	Strongly Agree	Strongly Agree
Overall, I am satisfied at the end of this course in terms of my expectations when I signed up for it	Strongly Agree	Agree	Strongly Agree
This course has helped me figure out more about my interests and possible future goals	Strongly Agree	Agree	Strongly Agree
I would recommend this course to someone else	Strongly Agree	Agree	Strongly Agree

Appendix D. Table 2: Themes and the frequency with which they were mentioned in open-ended questions from Section 3 of the PCQ (n=10) as well as feedback from the questions included on the final quiz (n=14). PCQ was taken by 10 of the 15 participants in program 1 (2018).

Theme	Mentions
**Positive Themes**	
Most memorable moments were consisted of excursions to the forest, river, or oxbow lake, daily night walks, tree-climbing training	10
Most memorable moment seeing sequence data come out of MinION or loading the sequencer	7
Have not heard of another course like this being offered	3
Enjoyed initial training on lab equipment, such as loading gels and using pipettes	2
Appreciate the opportunity to go from sample collection to sequencing in a portable lab space	5
Enjoyed project based learning based on real data	2
Enjoyed the balance between excursions and laboratory work	2
Feel that they acquired new or improved their skills in molecular lab work	14
Enjoyed hearing about conservation genetics applications of next generation sequencing from instructors' personal research and ITA's conservation efforts	4
Felt that course objectives and methods were clear and straightforward	8
**Things to be Improved**	
Expressed that the genetics "vocabulary" was overwhelming	7
Felt the course was at a level too advanced for their present education background	2
Felt that the course could have been better organized in terms of assigning participants final projects	1
Felt that the course was intense or laboratory work repetitive, with days longer than they would have preferred, ultimately suggesting a course extension of a couple of days	7
Expressed sharing the lab was a particular challenge, both due to space restrictions and navigating team work	9

Would have liked more time devoted to bioinformatics, including software provided in advance	8
Would have liked to have had more detailed protocols in advance to prepare better for each day	1
Would have liked to actually practice collecting samples from wildlife	2

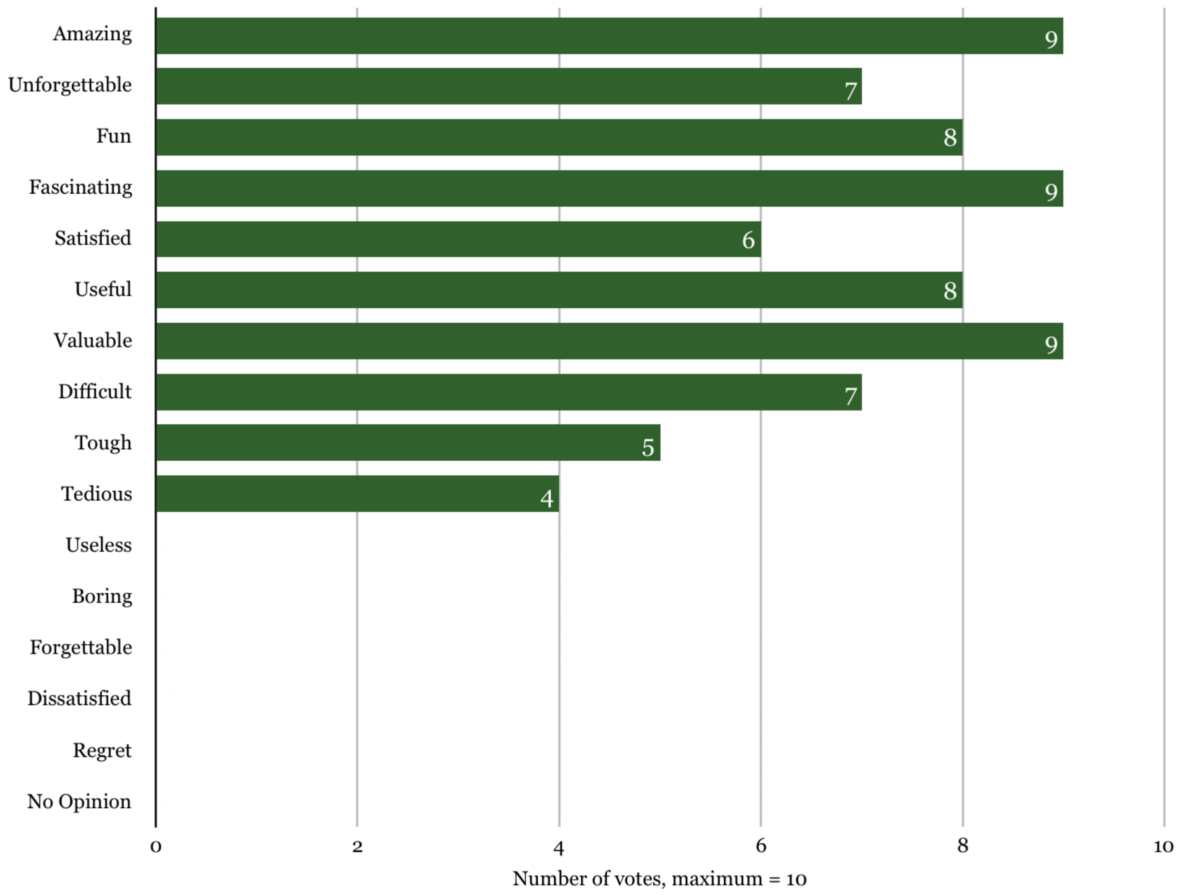

Appendix D. Figure 1: The number of votes received from course participants for positive markers (7), challenge-based markers (3), and negative markers (5) associated with taking this course. Maximum survey responses = 10. Survey taken by 10 of 15 participants in program 1(2018).

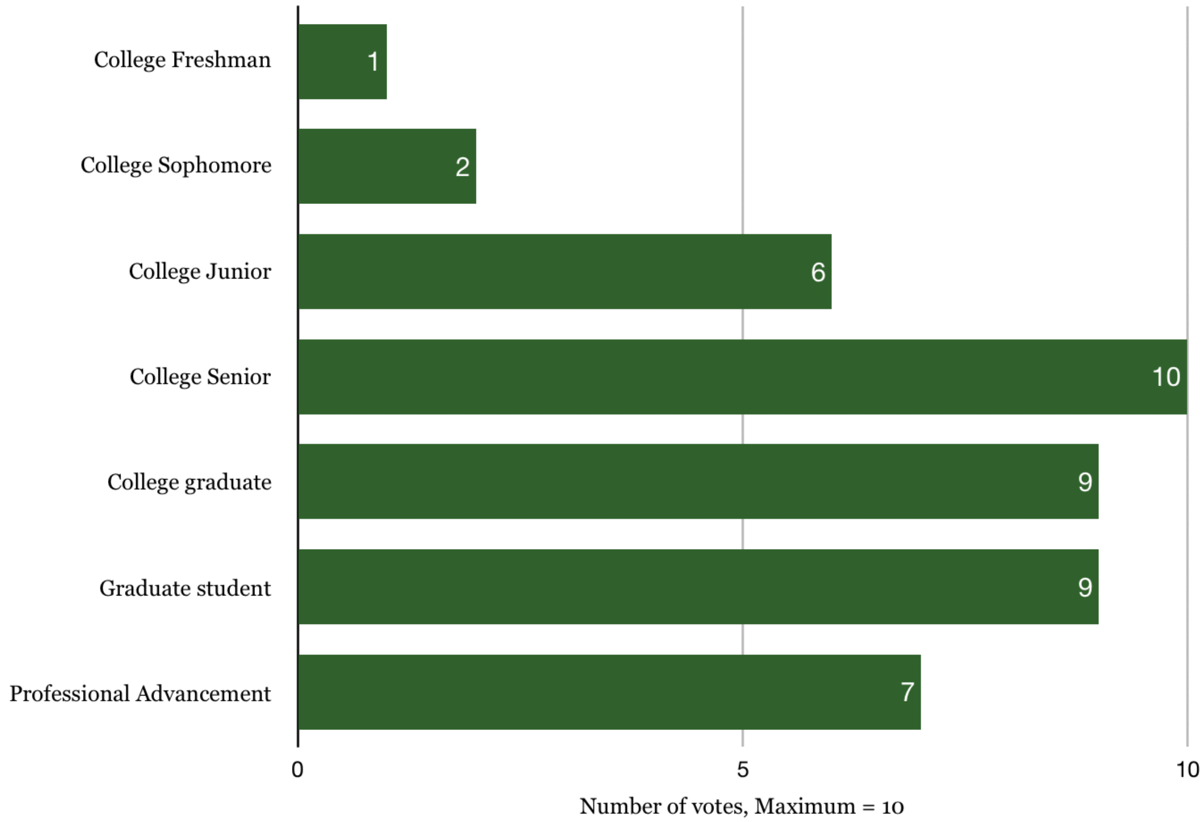

Appendix D. Figure 2: The number of votes received from course participants for each education category at which this course would be appropriate. Maximum survey responses = 10, survey taken by participants of program 1(2018).
