# Supplementary material for: Portable sequencing as a teaching tool in conservation and biodiversity research
Source: PLoS Biol. 2020 Apr 16;18(4):e3000667. doi: 10.1371/journal.pbio.3000667 (PMC7188297; doi:10.1371/journal.pbio.3000667)
Supplement: S2 Appendix — (DOCX) [file pbio.3000667.s002.docx]

**Appendix A, Table 1: Equipment in the Green Lab**

| **Item** | **Vendor** | **Catalogue Number/Model/Link** | **Number** |
| --- | --- | --- | --- |
| **Basic Infrastructure** | | | |
| Autoclave | TUTTNAUER | 2340M | 1 |
| Oven | Local |  | 1 |
| Microwave | Local |  | 1 |
| Lab Computer | Apple | Mac Mini | 1 |
| Refrigerator with small freezer | Local |  | 1 |
| Freezer | Local |  | 1 |
| Steam Water Distiller - 1 Gallon |  | Tuttnauer 9000 | 1 |
| AmScope 100X-1000X Trinocular LED Infinity Plan Phase Contrast Microscope |  | AmScope  T610-IPL-PH | 1 |
| UV Light | Amazon | [Link](https://www.amazon.com/gp/product/B00K6NUTRU/ref=oh_aui_detailpage_o03_s02?ie=UTF8&psc=1) | 1 |
| Voltage Converter | Diamond series | DSR-5000 | 1 |
| **Benchtop Equipment** | | | |
| Vortexer | FisherSci | Scientific industries Vortex-Genie 1 and 2 | 2 |
| pH Meter | Amazon | [Link](https://www.amazon.com/gp/product/B01N43WRY1/ref=oh_aui_detailpage_o01_s00?ie=UTF8&psc=1) | 1 |
| Mini Microcentrifuge | MiniPCR | [Link](https://www.minipcr.com/product/gyro-microcentrifuge-fixed-speed/) | 2 |
| Magnetic bead separation rack 1.5ml tubes | Ebay | [Link](https://www.ebay.com/itm/Magnetic-beads-separator-rack-for-Ampure-beads-made-for-1-5-mL-eppendorf-tubes/273014243549?hash=item3f90eac0dd:g:BZQAAOSwL9paUVkA) | 1 |
| Magnetic bead separation rack 96-well plate | Ebay | [Link](https://us.vwr.com/store/product/11630025/96-well-microtiter-plate-magnetic-separation-rack-new-england-biolabs) | 1 |
| Dry Bath Incubator | Used | Fisher Scientific 11-718 Dry Bath Incubator | 1 |
| Water Bath | VWR | [Link](https://www.ebay.com/itm/Hot-Water-Bath-VWR-Scientific-Digital-1285PC-900W-Laboratory-Benchtop-With-Lid-/272721444468) | 1 |
| 8-well PCR machine | MiniPCR |  | 5 |
| 16-well PCR machine | MiniOne |  | 2 |
| Bluegel Electrophoresis | MiniPCR | [Link](https://www.minipcr.com/product/bluegel-electrophoresis-with-built-in-illuminator/) | 3 |
| MiniOne Electrophoresis Package | MiniOne | [Link](https://theminione.com/product/minione-pcr-electrophoresis-package-i/) | 2 |
| Quantus Fluorometer | Quantus |  | 1 |
| Balance | Ebay | [Link](http://www.americaninstrument.com/equipment/product/?code=0682C-BALANCEBalances) Mettler  AJ100 | 1 |
| Microcentrifuge (24 well) | Hermle and Labnet spectrafuge 24D | [Hermle](about:blank)  Z 180 M  and Labnet | 2 |
| Cool Cube | Ebay | [Link](http://www.medicus-health.com/cool-cube.html) | 1 |
| Vacuum Pump | Millipore Sigma | WP6111560 | 1 |
| Large Centrifuge (Temp controlled) | Used | IEC Centra CL3 | 1 |
| MinION Sequencer | Oxford Nanopore Technology | [Link](https://nanoporetech.com/products/minion) | 1 |
| **Pipettes** | | | |
| Micropipette (20-200uL) | MiniPCR | [Link](https://www.minipcr.com/product/micropipette-set/) and [Link](https://theminione.com/product-category/micropipette/) | 6 |
| Micropipette (100-1000uL) | MiniPCR | [Link](https://www.minipcr.com/product/micropipette-set/) and [Link](https://theminione.com/product-category/micropipette/) | 6 |
| Micropipette (1-10uL) | MiniPCR | [Link](https://www.minipcr.com/product/micropipette-set/) and [Link](https://theminione.com/product-category/micropipette/) | 6 |
| Multichannell pipette 20-200ul | Ebay | [Link](https://www.ebay.com/p/Rainin-Pipet-lite-L200-LTS-8-Multi-Channel-Pipette-Adj-20-200-UL-CALIBRATED/1134189290) and [Link](https://www.amazon.com/gp/product/B0062A1JMK/ref=oh_aui_search_detailpage?ie=UTF8&psc=1) | 2 |
| Pipette pumps (2ml, 5ml, 10ml) | Amazon |  | 1 set |

**Appendix A, Table 2: Recommended Items for a Pop-up Laboratory for Field Conservation Genomics Training Programs for 12 Participants**

| **Item** | **Vendor/Model** | **Notes** | **Unit cost (USD)** | **Number of units** | **Total cost (USD)** |
| --- | --- | --- | --- | --- | --- |
| **Basic Infrastructure***** | | | | | **$1750** |
| Autoclave | Eg: TUTTNAUER 2340M | Alternatively, bring in pre-sterilised plastics | $500 | 1 | $500 |
| Oven | Local | This helps dry autoclaved materials | $200 | 1 | $200 |
| Microwave | Local | Primarily for agarose gel prep. Alternatively, purchase pre-made gels. | $50 | 1 | $50 |
| Refrigerator with small freezer | Local | Critical to store flowcells and other laboratory ingredients. However, flowcells can survive at RTP for 1 month, and its possible to obtain PCR and library prep-supplies in lyophilised formats too. | $300 | 1 | $300 |
| Steam Water Distiller - 1 Gallon | Tuttnauer 9000 | Alternatively, purchase clean water directly. | $500 | 1 | $500 |
| Voltage Converter | Diamond series DSR-5000 | Critical if some equipment has different electrical requirements than the habitat country. | $200 | 1 | $200 |
| **Alternatives to Large Equipment** | | |  |  | $242 |
| Autoclaved plastics | Assorted | Pipette tips, tubes, and other plastics | $200 | 1 | $200 |
| Clean water | [G-Biosciences](https://www.gbiosciences.com/Water-Molecular-Grade?utm_campaign=Shopping+Campaign&utm_term=&utm_source=adwords&utm_medium=ppc&hsa_src=g&hsa_ver=3&hsa_cam=1628359862&hsa_kw=&hsa_ad=311528445109&hsa_tgt=pla-294682000766&hsa_mt=&hsa_acc=6752996364&hsa_grp=60502138925&hsa_net=adwords&gclid=CjwKCAiAi4fwBRBxEiwAEO8_HnAcvPNMN6TLYbW5d03d8FEdldj3ppiNR6GyQJyyk61EbK1s4zeaXBoCJNgQAvD_BwE) | 500 mL bottle of moleculsr grade water | $21 | 2 | $42 |
| **Benchtop Equipment** | | | | | **$7349** |
| Vortexer | Benchmark Scientific Vornado Mini Vortexer | Generic vortexes also cheaply available second-hand, but can be heavy. Alternatively, can manually homoegenise materials by inverting by hand. | $190 | 2 | $380 |
| Mini Microcentrifuge | [MiniPCR](https://www.minipcr.com/product/gyro-microcentrifuge-fixed-speed/)/[Mini One](https://theminione.com/product/the-minione-centrifuge/) | [Alternatively, use the paperfuge](https://www.biorxiv.org/content/biorxiv/early/2016/08/30/072207.full.pdf); price listed for cheaper fixed speed from MiniPCR | $160 | 2 | $320 |
| Magnetic bead separation rack 1.5ml tubes | Ebay | For bead-based purification | ~$30 | 1 | $30 |
| Optional: Incubator | Dry or Water bath, thermomixer | For short incubations, can use a PCR machine; Note: MiniPCRs cannot hold below 30C | N/A | 1 | N/A |
| PCR Machines | [MiniPCR](https://www.minipcr.com/product-category/equipment-and-accessories/minipcr-thermal-cycler/)/[MiniOne](https://theminione.com/product/minione-pcr-system/) | 8- or 16-well devices; MiniPCR doesn’t have peltier technology, making it slower to switch temperatures, but MiniOne devices are possibly less rugged for including it  Option 1 (no Peltier): 2 x Mini-16 ($795)+ 1 Mini 8 (650) (40 wells)  Option 2 (Peltier): 3 x MiniOne (48 wells) ($799) + 3 x Kindle controllers ($99) | $2240  $2694 | 1  1 | $2240  $2694 |
| Electrophoresis Machines + Imagers | [MiniOne](https://theminione.com/product/minione-system/) | Image clarity unmatched, portable and rugged devices, use agarose gels. | $279 | 3 | $837 |
| Fluorometer | Quantus | This is expensive but necessary to normalise before pooling. The one area in which a low-cost alternative doesn’t exist to date. Includes 500 assays, some tubes | $1872 | 1 | $1872 |
| Cool Cube | [Assorted vendors](https://www.pipettes.com/coolcube-microtube-and-pcr-plate-cooler.html?ff=1&fp=4756&utm_source=Google+Shopping&utm_medium=Ads&utm_campaign=Feed&utm_term=&utm_campaign=IM+-+Shopping+-+SPAG&utm_source=adwords&utm_medium=ppc&hsa_acc=9371089172&hsa_cam=705805399&hsa_grp=35651571086&hsa_ad=162205438229&hsa_src=g&hsa_tgt=pla-402662657499&hsa_kw=&hsa_mt=&hsa_net=adwords&hsa_ver=3&gclid=CjwKCAiAi4fwBRBxEiwAEO8_Hj138l-GC-6u-RD5_m3OzbCv-jYbrOa809eWuvMO4MRBzB_1h9HAqRoCeW8QAvD_BwE), check ebay also | Alternatively, freeze water in the lid of a PCR tube tray for a makeshift cool cube | $110 | 2 | $220 |
| MinION Sequencer | Oxford Nanopore Technology | Comes with 2 flowcells in a starterpack, or 12 Flongle flowcells ($1800 extra). Check wait times for Flongle production. | $1000 | 1 | $1000 |
| Pipettes | MiniOne/MiniPCR | Affordable and accurate devices; alternatively, use fixed volume pipettes for an even cheaper alternative; MiniOne set of 3 ($195); MiniPCR ($150) | $150 | 3 | $450 |
| Total Overall Cost | | | | | $7591 |

*** Basic infrastructure is highly cost variable based on where the program takes place. Also, much of it could be replaced with single-use consumables. Thus, we exclude these costs from the overall total.

Note: Where possible, cheaper alternatives are included. Prices are based on an estimated level of convenience for 12 participants to work together. If class-time is longer, fewer devices could be shared. It is also possible to package certain items together for further discounts. For eg: gel electorphoresis devices + PCR machines.

**Appendix A, Table 3: Ingredients Lists for Training Program for 12 Participants Using DNA Barcoding as a Case Study.**

| **Item** | **Vendor** | **Notes** | **Unit cost (USD)** | **Number** | **Total cost (USD)** |
| --- | --- | --- | --- | --- | --- |
| Taq polymerase (dNTPs and Magnesium included) | Promega - M7406, 100u, GoTaq® G2 Hot Start Polymerase | Cheaper Taqs available from MiniOne/MiniPCR but quality less reliable. For simpler teaching use master mixes although we have found that fundamentals are better understood when each PCR ingredient is used separately. | $60 | 2 | $120 |
| Quantus Fluorometer Assays | Promega | One set is more than sufficient for one field course (~500 assays), included in starter pack | N/A | 1 | N/A |
| PCR strip tubes | Any | Avoid plates since PCR machines are small. Unit Price for 100, 0.2uL tubes | $12 | 5 | $60 |
| 1.5mL Microcentrifuge Tubes | Any | Simplest for a variety of purposes. Unit price per 500 | $27 | 2 | $54 |
| Barcoding Kit | [Oxford Nanopore Technology](https://store.nanoporetech.com/pcr-barcoding-expansion-1-12.html) | Available in 12 and 96 barcode formats, price here for 12. (Alternatively, use custom barcodes as a cheaper alternative) | $288 | 1 | $288 |
| Gelgreen | [MiniOne](https://theminione.com/product/gelgreen-stain-50-µl/)/ [MiniPCR](https://www.minipcr.com/product/gelgreen-nucleic-acid-gel-stain-10000x-in-water-200µl/) | Product can be stored at rtp or refrigerated; a tube of 50uL more than enough for one field course. | $20 | 1 | $20 |
| Gel loading dye | [MiniOne](https://theminione.com/product/5x-sample-loading-dyes-orange-g/)/ [MiniPCR](https://www.minipcr.com/product/gel-loading-dye-blue/) | One tube sufficient for one course | $10 | 1 | $10 |
| Agarose | Any | [MiniOne](https://theminione.com/product/agarose-25g/)/[MiniPCR](https://www.minipcr.com/product/agarose-electrophoresis-grade-20-gr/) also sell loose agarose, pre-measured tablets, or pre-mixed gel cups; unit price for 20g | $40 | 1 | $40 |
| TBE Buffer 10X | [MiniOne](https://theminione.com/product/tbe-running-buffer-concentrate/), [MiniPCR](https://www.minipcr.com/product/20x-tbe-electrophoresis-buffer-250-ml/) | Best to purchase this pre-mixed at 10X concentration; 500mL | $18 | 1 | $18 |
| Primers | IDT or other | Depending on markets required, purchase from vendors in bulk, lyophilised. | $20 | 2 | $40 |
| Gel loading and micropipetting practice kit | [MiniPCR](https://www.minipcr.com/product/micropipetting-101/) | Easy to use, reusable and affordable kits that help with pipette-use training and refreshers; one kit = 32 uses. | $54 | 1 | $54 |
| Spin-columns | Epoch LifeSciences, [link](http://www.epochlifescience.com/Product/SpinColumn/minispin.aspx) | Cheap and good quality spin-columns of different sizes for DNA extraction and purification; 250 spin columns | $95 | 1 | $95 |
| Total overall cost | | | | | $799 |
