# Supplementary material for: Portable sequencing as a teaching tool in conservation and biodiversity research
Source: PLoS Biol. 2020 Apr 16;18(4):e3000667. doi: 10.1371/journal.pbio.3000667 (PMC7188297; doi:10.1371/journal.pbio.3000667)
Supplement: S3 Appendix — (DOCX) [file pbio.3000667.s003.docx]

**Appendix B: Sample Program Syllabus**

[Hosting Institution] [Laboratory/Field Site Location]

[Program Title]

Program Syllabus

**Program Introduction**

Biological research is turning to genetic research methods for a deeper look into the biological factors that encode behavior and physiology. We can use genetic techniques to determine species delimitations, define populations, understand mating systems, explain behavioral differences in foraging efficiency, screen for disease, conduct paternity studies, evaluate immune status and functioning, and explore microbiome diversity… and these are just a few examples of the full breadth of the field as applied to wildlife biology. When viewed through the additional lens of conservation, genetics and genomics methodologies are assisting in biomonitoring through DNA barcoding, exploration of environmental DNA, and detection of rare species from pooled community samples.

In the past few years we have even witnessed the successful deployment of instruments that enable molecular work to be conducted ‘on-the-fly’ and in the field. These new tools are minimizing the hassles and barriers associated with transporting samples around the world to distant labs that possess the equipment and resources to extract, amplify, and sequence DNA. In many ways, this new technology is democratizing wildlife research by empowering field scientists all around the world with genetic tools to directly advance their research and conservation initiatives.

This program will take students to the [country/habitat], where they will learn how field research is conducted, assist in sample collection, and then extract, amplify, sequence, and interpret genetic data to answer several practical research questions about wildlife ecology and biodiversity. Participants will go from sample collection to sequence analysis directly at the field site, which is the science of the future. This program will provide an introduction to next-generation sequencing technology to biologists, who will gain not only the skills requisite for field research but the technical know-how to employ genetic research tools in the field.

**Case Studies**

In this course, we will focus on three specific cases (class size and time permitting) in which cutting-edge genomics can help us solve mysteries common to wildlife research in the field. The ultimate goal of all of these projects will be to use the MinION, a USB-sized powerful sequencer that is revolutionizing how we do genomics in some of the most remote places on the planet.

***Case 1: Biodiversity Screening with DNA Barcoding***

DNA barcoding, or the science of sequencing a common marker across multiple species to assist with species identification, is slowly but surely adapting to using high-throughput sequencing in lieu of single-amplicon Sanger sequencing. However, many of the least-known species are living in highly threatened habitats across the globe in which biological specimens are highly protected to ensure the conservation of these species. This makes them hard to access by large sequencing facilities, thus increasing the gap between places that require biomonitoring and people who can provide such services using genetic methodologies.

Using portable and relatively low-cost devices, we can bring the science directly to the sample, and circumvent expensive and often unsuccessful attempts to export biological specimens. In this case study, we will collect [list samples and species of interest] and use [list markers] to barcode the DNA of each species, and compare it to a reference database. In some cases, where species have been sequenced before, we expect to find matches to public reference databases; but in others, we might contribute the first sequences for these species to the public.

Skillsets: Sample collection from [list sources], sample storage and sterile technique, DNA extraction using multiple kits for comparison, amplification of barcoded regions in the genome, gel electrophoresis to confirm amplicon sizes, sequencing of the regions using a MinION

***Case 2: Exploring diet and gut flora***

A microbiome is a collection of the genes of all the microbes in a community, and a disturbance of the “normal” microbiome of the gut of an animal (including humans) can result in disease. However, what is normal? Which microbes are present and what do they do? Although much of this information is now known for humans, it is completely unknown in many wild animals.

For this case study, we will collect fecal samples from [list sources, we recommend using local wildlife rehabilitation centers if wild samples are not available]. Our goal is to conduct 16S sequencing of all of the bacteria present in these samples.

Skillsets: Sample collection (wild/captive), storage and sterile technique, DNA extraction, PCR amplification of the genetic material in the 16S ribosomal subunit, multiplexing of samples and cDNA library prep for sequencing on the MinION, subsequent analysis of data using the WIMP workflow.

***Case 3: Environmental DNA***

There are two ways in which environmental DNA screens are typically carried out. First, we could target a specific species - for e.g. an invasive species or a rare/cryptic species - and try to detect traces of its DNA in the environment. Alternatively, we could try to identify all the species in a community at the same time. For both of these projects, we begin by collecting environmental samples, typically water from a lake or stream or a soil sample (but this could include leaf litter or sediment), and filtering out biological materials onto a filter paper. DNA is then extracted from the residue on the filter paper and screened either for a specific species’ DNA or for a more general marker that could then be compared to databases to identify all molecular operational taxonomic units (MOTUs). Some alternative sources of environmental DNA include bulk samples, such as a pooled sample of all the insects collected in a Malaise trap, or a fecal sample, which is a collection of a multitude of organisms, each with their own unique trace of DNA.

In this case study, we will use [list sample sources] with [list markers] to examine environmental DNA using a MinION sequencer. We can use ONT’s WIMP platform to identify MOTUs using existing reference databases.

Skillsets: Sample preparation and collection, DNA extraction, library preparation, sequencing on the MinION and analysis using WIMP.

***We will try to answer a few additional questions with all case studies:***

- Can we use the What’s In My Pot (WIMP) workflow to accurately classify these species to a reference database in real-time for all of the case studies?
- How many different kinds of samples can we multiplex at one go on a MinION?
- Does the choice of DNA extraction kit affect the outcome?
- Does the length of time we let the MinION run for affect the accuracy of our species identifications?

**Course Objectives**

The goals of this course are to give participants advanced training in field techniques important to the collection of biological samples from wildlife, their prey and their parasites, all the way to sequencing DNA from these sources.

The course has the following broad objectives:

* To engage in both independent and team-based data collection

- To teach sample collection techniques from [describe wildlife]
- To learn sample storage and clean-lab protocols in the field
- To extract DNA in a field laboratory
- To test DNA quality and quantify it
- To run basic PCRs for a range of markers using multiple protocols on field PCR machines (smaller, lighter, lower-scale and more rugged than typical lab-based machines)
- To explore metagenomics in the field using the above case studies

**Table 1: Course Topics**

| **Topic of Study** | **Activity** | **Description** |
| --- | --- | --- |
| **I. Introduction** |  |  |
| > Threats to the local habitat; conservation efforts of local organisations | Lecture | A review of the major conservation approaches in the [habitat], including the conservation and research efforts of [NGOs, government, etc.] |
| > Field ethics, safety precautions, rules,and useful tips. | Discussion | Keeping your footprint to a minimum while working with wildlife in the tropics, and ensuring your safety and that of the wildlife around you. |
| > DNA sequencing and genomics | Lecture/Discussion | An introduction to genomics and its practical applications in the field |
| > Genomics and Ethics | Discussion | What should we be thinking about when applying genomics to wildlife research? Whose rights come into question? Do different countries regulate genomics in different ways? |
| **II. Navigation and Space Use** | |  |
| > Basic functions of a handheld GPS and compass | Demonstration | Getting familiar with the most important pieces of equipment you will have in the field. |
| > Waypoint and track data and how to use them | Exercise | Recording key features of the research station with waypoints and tracks |
| > Visualizing spatial data | Exercise | Manipulation of GPS data; creating a digital field map (laptop with USB port required for each participant) |
| **III. Collecting Biological Specimens** | | |
| > Field methodology: indirect observation and biological sampling | Lecture/Practical Exercise | Tracking [eg wildlife], identifying plants based on botanical features, and collecting non-contaminated samples from each. |
| > Field methodology: sample storage and preparation | Lecture/Practical Exercise | Sample collection methods in the field, time-scales for DNA deterioration, and field laboratory sterile technique |
| **Topic of Study** | **Activity** | **Description** |
| **IV. Basic Field Laboratory Techniques** | |  |
| > Basic laboratory techniques | Practical Exercise | Regardless of participant background, we will spend a moment practicing good pipetting techniques, sample volume calculations, and gel loading techniques. Repetition is key, getting everyone onto the same skill level |
| > Laboratory Safety | Lecture/Practical Exercise | Even in the field, lab safety is critical. We will go over protocols, cautionary tales, and specific examples of how not to hurt yourself in a field laboratory. |
| > Lab recipes | Practical Exercise | We will learn the processes behind the various lab protocols or recipes we will be following in this course |
| **V. Genomics in the Jungle** | | |
| > DNA barcoding | Lecture | The history of species identification using barcodes, including some of the most exciting applications in wildlife science |
| > Environmental DNA | Lecture | DNA is everywhere, in everything - so why would we care about picking up trace DNA? What are the useful applications of this technique? |
| > Microbiome analyses | Lecture | Microbiome analyses can tell us a great deal about human health - can the same be said for animals? What is a normal monkey gut even look like? |
| **VI. Genomics in a Jungle Lab** | |  |
| > DNA extraction | Practical Exercise | Whole genome amplifications and genomic DNA extraction |
| > DNA quantification | Practical Exercise | How much DNA do you have? |
| > PCRs | Practical Exercise | Amplifying markers for all three case studies using PCRs in a field laboratory |
| > Gel electrophoresis | Practical Exercise | Testing if your PCR worked using gel electrophoresis and PCR product quantification |
| > Library prep | Practical Exercise | Creation of DNA libraries from samples |
| > Multiplexing and indexing | Practical Exercise | Minimising sequencing costs by running multiple samples in a single run - multiplexing and indexing samples to tell them apart afterwards |
| > Using the MinION | Practical Exercise | Learn to run sequences on a MinION device - cutting edge genetic sequencing using nanopore flowcells |
| **Topic of Study** | **Activity** | **Description** |
| > Basic bioinformatics | Practical Exercise | How to interpret data from the MinION - both real-time and post-hoc |
| **V. Excursions and Activities (time and weather permitting); examples relevant to Peruvian programs** | | |
| > Canopy Walkway | Excursion | Participants will be given the opportunity to climb and view the Amazon rainforest from network of rainforest canopy walkways. |
| > Claylicks | Excursion | We will visit Lake Sandoval to view the famous macaw and mammal claylicks on site |
| > Taricaya Rehabilitation Center | Excursion | The illegal wildlife trade is blooming in Peru, and rehabilitation centers are the final stop for those animals lucky to be recovered - but is being in captivity changing them irrevocably? |
| > Guest Lectures | Lecture | This is a popular location for field researchers. Participants will be given the opportunity to be exposed to various research groups and interests during their time here, including projects that directly emphasize wildlife biology. |

**Table 2: Daily Schedule**

| **Date** | **Morning** | **Afternoon** | **Night** | **Reading Discussion** |
| --- | --- | --- | --- | --- |
| **Day 1** | **Arrive on site;** Go directly to the station | Get to know your peers and instructors | Get to know your peers and instructors | *Discussion:*  Field ethics, safety, rules, and tips |
| **Day 2** | Map the trails: a navigation exercise | Welcome to the Lab  Lab safety protocols  A lab skills refresher | *Lecture*  Introduction to the region, organisations working in the area, conservation and threats that the area faces. | **Papers**: Swenson et al, 2011; Swamy et al., 2011 |
| **Day 3** | **Group 1:**  Field Excursion  **Group 2**  Sample storage in the field  Recipes for success  DNA extraction lab 1 (overnight lysing) | **Group 1:**  Sample storage in the field  Recipes for success  DNA extraction lab 1 (overnight lysing)  **Group 2 :**  Field Excursion | *Lecture*  Genetics and wildlife biology | *Discussion*: Lab reports and formats. Group assignments  **Paper**: Zeng and Martin, 2017; Erwin 1982 |
| **Day 4** | **Group 1**  Lab: Complete overnight extractions  **Group 2**  Field Excursion | **Group 1**  Field Excursion  Navigating off trail and field sampling  **Group 2**  Lab: Complete overnight extractions | *Lecture*  *Pre-dinner*: Genomics and wildlife biology  *Post-dinner*: Introduce the Case Studies. | Digital Lab Notebook: Due at the end of the night. |
| **Date** | **Morning** | **Afternoon** | **Night** | **Reading Discussion** |
| **Day 5** | **Field Sampling Activity**  Quiz 1 | FTA extractions | *Lecture:* Conservation Genetics | *Discussion*: The wildlife trade in Peru |
| **Day 6** | **All Day:**   - Split into lab teams - PCR setup and amplifications for various markers - Field activities while PCRs are running - PCR cleanups | | *Protocol Troubleshooting and Planning* | **Paper**: Chiou et al., 2018 |
| **Day 7** | **All Day:**   - Continue lab work from Day 6 - Protocol troubleshooting and planning | | | **Paper**: Thrasher et al., 2018 |
| **Day 8** | - Barcoding PCRs for Multiplexing - PCR cleanups - Normalising Samples - Pooling into Libraries | |  | **Paper**: Cusco et al., 2017 |
| **Day 9** | Continue lab work from Day 8  Quiz 2 | |  | **Paper**: Krehenwinkle et al., 2018; Pomerantz et al., 2018 |
| **Day 10** | - Library prep - Trail hikes in between lab - End the day by starting MinION runs | | Real-time results with the MinION (!!) | Digital Lab Notebook: Due when library prep is done for your project. |
| **Date** | **Morning** | **Afternoon** | **Night** | **Reading Discussion** |
| **Day 11** | - Examining real-time sequence results - Exploring MinION outputs - Complete all MinION runs for projects | | Real-time results with the MinION |  |
| **Day 12** | Data analysis with Case Studies | Time to practice presentations and prep for final | Turn in Draft 1 of your section of the course paper | Final quiz: |
| **Day 13** | Final excursions and hikes  Completing your projects  Preparing for presentation | Time off to pack | *Final presentations: 10 mins per group* |  |
| **Day 14** | **Depart from the field station** | | |  |

**Course Work**

*Lab notebook (250 pts)*

Paper notebooks in a lab are soon becoming a thing of the past. We will use the Benchling software that is both free and flexible online, to conduct all the exercises in the course. On the Benchling account for the course will be all lab protocols and templates, pre-designed and ready for use. Participants will simply choose the appropriate template and store all data online. At the end of the course, your Benchling lab notebook will form a part of your grade, as maintaining an accurate record of the work you do in a lab is a crucial part of being a good research scientist.

*Sample List (100 pts):*

All participants will contribute towards the course sample list, documenting a range of metadata for each sample that will help us identify it in the future. The ongoing sample list will be judged as a group at the end of the course and individual effort to locate and collect samples will also be appreciated.

*Lab Exercises (250 pts):*

Every case study is going to involve a series of lab sessions, each of which will have reports due at the end. These may be recorded on Benchling or turned in by hand, depending on the exercise. Your ability to complete the exercises, remain enthusiastic and a good team player will determine your grade for each lab exercise.

*GPS Mapping (100 pts)*

Several activities will involve recording GPS data and manipulating it in the Garmin Basecamp program, which is available for free download. At the end of the course, students will turn in the data from these exercises, which will include tracks made during off-trail hikes and wildlife follows, as well as a student-generated map of the field station.

**General Reading List**

| *Pomerantz, A., Penafiel, N., Arteaga, A., Bustamante, L., Pichardo, F., Coloma, L., Barrio-Amoros, C., Salazar-Valenzuela, D., Prost, S.* (2018). **Real-time DNA barcoding in a rainforest using nanopore sequencing: opportunities for rapid biodiversity assessments and local capacity building.** GigaScience 7(4), 74 14.<https://dx.doi.org/10.1093/gigascience/giy033> |
| --- |
| [*Krehenwinkel H., Pomerantz A., Prost, S..* **Genetic Biomonitoring and Biodiversity Assessment Using Portable Sequencing Technologies: Current Uses and Future Directions.** Genes. 2019. p. 858. https://doi:](http://paperpile.com/b/uAwo9X/Jg5b)[10.3390/genes10110858](http://dx.doi.org/10.3390/genes10110858) |
| *Mezzasalma, V., Bruni, I., Fontana, D., Galimberti, A., Magoni, C., Labra, M.* (2017). **A DNA barcoding approach for identifying species in Amazonian traditional medicine: The case of Piri-Piri** Plant Gene 9 (J. Ethnopharmacol. 76 2001), 1 5.<https://dx.doi.org/10.1016/j.plgene.2016.11.001> |
| *Janjua, S., Fakhar-I-Abbas, William, K., Malik, I., Mehr, J.* (2016). **DNA Mini-barcoding for wildlife trade control: a case study on identification of highly processed animal materials** Mitochondrial DNA Part A 28(4), 1-3.<https://dx.doi.org/10.3109/24701394.2016.1155051> |

*Bohmann, K., Evans, A., Gilbert, M., Carvalho, G., Creer, S., Knapp, M., Yu, D., Bruyn, M.* (2014). **Environmental DNA for wildlife biology and biodiversity monitoring.** Trends in Ecology & Evolution 29(6), 358-367.<https://dx.doi.org/10.1016/j.tree.2014.04.003>
