# Supplementary material for: Portable sequencing as a teaching tool in conservation and biodiversity research
Source: PLoS Biol. 2020 Apr 16;18(4):e3000667. doi: 10.1371/journal.pbio.3000667 (PMC7188297; doi:10.1371/journal.pbio.3000667)
Supplement: S4 Appendix — (PDF) [file pbio.3000667.s004.pdf]

## Appendix C: Laboratory protocols and bioinformatics pipelines for case studies.

In this appendix, we outline laboratory protocols, bioinformatics pipelines, and suggested references for three of the most commonly applied case study topics outlined in the manuscript (Table 1):

- DNA barcoding
- Metabarcoding
- eDNA

First, however, we suggest perusing the next section on general bioinformatics guidelines to prepare participants to successfully complete the bioinformatic pipelines used in these case studies.

# Bioinformatics Basics:

If reliable internet is available at the site of the program, it is also possible to utilise an online teaching tool called DNA Subway (https://dnasubway.cyverse.org). DNA Subway allows the upload of up to 100kb of data for analysis on five color-coded subway lines. The red line allows for genome sequence annotation, finding genes and identifying them via BLASTN and BLASTP search algorithms. The yellow line allows an input of 10Kb of a gene or protein to find related genes/proteins. The blue line allows for the analysis of phylogenetic relationships between sequences. The green line takes NGS data as input and assists with transcriptome analyses. Finally, the purple line facilitates microbiome analyses, including clustering and alpha/beta diversity calculations. All of these tools, while extremely useful to illustrate various bioinformatic processes with practice datasets, were not designed to accommodate nanopore sequencing data. Therefore, we provide below the basic commands required to analyse nanopore data locally, while in the field.

Most tools designed for nanopore data execute cleanly using bash in a Terminal window on Mac or Unix Operating Systems. However, for those with Windows machines, we suggest using Windows 10, which includes a Linux-like UNIX platform.

We recommend all participants spend a moment prior to the training program familiarizing themselves with the basics of bash scripting. One useful tutorial online that could be completed prior to the program is Bash Academy's Introduction to Bash Scripting (available here). There are many other resources available online that can assist participant understanding of the bash shell script, but the goal of this program is to gain fluency in executing the following commands:

**Appendix C, Table 1: Common bash commands and their uses. For a more detailed list, see here.**

<table>
<tr><th>Command</th><th>Usage</th><th>Modifications</th></tr>
<tr><td>pwd</td><td>Print working directory</td><td></td></tr>
<tr><td>cd</td><td>Change directory</td><td>cd .. ; cd ~</td></tr>
<tr><td>ls</td><td>Listing files in a directory</td><td>ls -lah, -la</td></tr>
<tr><td>-h</td><td>Getting help for a particular tool</td><td></td></tr>
<tr><td>man</td><td>Reading the manual for a particular tool</td><td></td></tr>
<tr><td>nano</td><td>Reading and editing simple text files within the terminal window</td><td></td></tr>
<tr><td>head/tail/less</td><td>Viewing portions of a file within the terminal window</td><td></td></tr>
<tr><td>cp/mv</td><td>Copy or move files to a new location</td><td></td></tr>
<tr><td>cat</td><td>Concatenate files together to form a new file</td><td>-r</td></tr>
<tr><td>mkdir</td><td>Make a new directory</td><td></td></tr>
<tr><td>rm/rmdir</td><td>Delete a file or a directory</td><td></td></tr>
<tr><td>| / > / <</td><td>Redirect output to a file, or pipe output from one command into another</td><td></td></tr>
<tr><td>echo / printf</td><td>print output to a file</td><td></td></tr>
<tr><td>gunzip/gzip/tar</td><td>Learn how to work with zipped files; useful for many tool installations and for working with large datafiles</td><td></td></tr>
<tr><td>brew/pip install/wget</td><td>Downloading and installing tools within the terminal window</td><td></td></tr>
<tr><td>sudo/chmod</td><td>Change file user permissions to make scripts or tools executable</td><td></td></tr>
<tr><td>grep/awk*</td><td>*Optional at this level, but useful to write custom functions</td><td></td></tr>
<tr><td>Control-c</td><td>Terminates a process</td><td></td></tr>
</table>

The Workflow

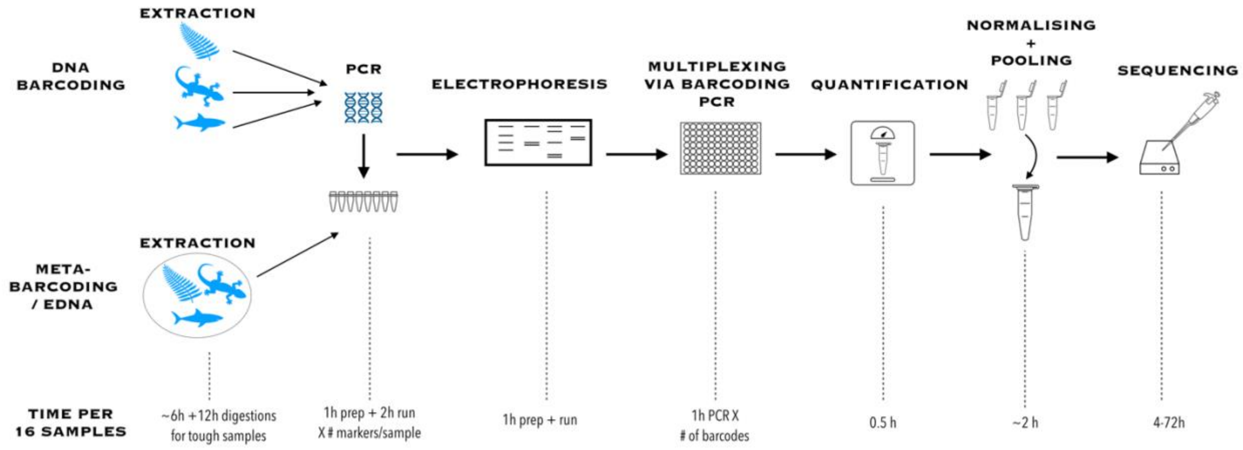


Figure 1. An overview of the three cases outlined in this appendix with notes on the duration of each step of the workflow for a set of 16 samples.

*A note on application to aquatic environments:* Although these protocols were developed for use in terrestrial systems, they are readily applied to samples acquired from aquatic or marine environments. The primary differences lie in collection protocols. Below, find some resources on specifically aquatic systems.

**Suggested Readings**

| **Resource** | **Reference** |
|---|---|
| For a summary of DNA recovery from aquatic environments, this is a great review. | *Rees, H.C., Maddison, B.C., Middleditch, D.J., Patmore, J.R. and Gough, K.C.,* 2014. The detection of aquatic animal species using environmental DNA–a review of eDNA as a survey tool in ecology. *Journal of Applied Ecology*, *51*(5), pp.1450-1459. |
| Metabarcoding in aquatic systems | *Deiner, K., Fronhofer, E.A., Mächler, E., Walser, J.C. and Altermatt, F.,* 2016. Environmental DNA reveals that rivers are conveyer belts of biodiversity information. Nature communications, 7, p.12544. |
| Preservation condition effects on sampling in neotropical rivers | *Sales, N.G., Wangensteen, O.S., Carvalho, D.C. and Mariani, S.,* 2019. Influence of preservation methods, sample medium and sampling time on eDNA recovery in a neotropical river. Environmental DNA. |

## Sources of Error

Equipment failure: During our programs, we experienced some perplexing PCR failures. Despite re-running reactions repeatedly, these did not resolve. We then discovered that several of the laboratory's transilluminators were not bright enough to reveal clear gel bands. Eventually the malfunctioning devices were replaced, and this glitch provided an opportunity for participants to learn to troubleshoot unexpected results. It is always wise to have more than one piece of equipment and to be particularly conscious of power surges under field conditions that could result in equipment malfunctions.

Sample contamination: Given field conditions, achieving a high standard of sterility may not be feasible. We recommend using portable or pop-up hoods if possible, but if not, we use a decontamination protocol consisting of 10% bleach, two distilled water rinses, and then 70% ethanol. This protocol can be used to decontaminate surfaces, pipettes, or scalpels/tweezers that come into contact with multiple samples. We also recommend setting aside one set of pipettes and other benchtop equipment (if possible) for extractions alone, to minimize contamination of PCRs. Including suitable positive and negative controls in all experiments is strongly advised.

Sequencing conditions: The MinION is a device that is easily powered off a laptop, but it does have a fairly narrow window of functionality when it comes to temperature. In warm and humid conditions, it is advisable to have an ice pack that the device can be placed on to reduce running temperatures so that sequencing can begin. It is also important that the surface on which the device is placed be level – this assists in the correct loading of the flowcell without the library overflowing any of the exit ports.

## DNA Barcoding

**Goal**: To multiplex sequence amplicons of universal barcoding markers across a range of taxa.

**Recommended Samples**: It would be ideal to include a range of taxa and tissue types to familiarise participants with different extraction protocols and barcoding markers. Common plant markers include *matk, rbcl* and *trnH-psbA intergenic spacer*, while animal markers include *cytochrome b* (*cytb*) and *cytochrome oxidase subunit 1* (*COI*). For a full list of markers for a wide variety of tissue types see [1].

**Ingredients**:A full ingredient list is linked to the protocol below (also available at protocols.io.dx.doi.org/10.17504/protocols.io.y2sfyee) but in general terms includes:

- A DNA extraction kit
- Or in-house extraction buffers [see 1] + spin columns
- Primers
- PCR master mix or taq+dNTPs
- Barcoding kit or custom barcodes
- dA-tailing and end-repair kit (New England Biolabs)
- Library prep kit (ONT)

# DNA Barcoding in a Field Setting

Mar 11, 2019

Aaron Pomerantz[1,2], Mrinalini Watsa[3,4,5], Stefan Prost[6,7], Gideon Erkenswick[3,5]

[1]University of California, Berkeley, CA, USA, Department of Integrative Biology, [2]Marine Biological Laboratory, Woods Hole, MA - USA, [3]University of Missouri - Saint Louis, [4]Washington University in Saint Louis, [5]Field Projects International, [6]5 LOEWE-Center for Translational Biodiversity Genomics, Senckenberg Museum, 60325 Frankfurt, Germany, [7]South African National Biodiversity Institute, National Zoological Garden, Pretoria 0184, South Africa

1 Works for me dx.doi.org/10.17504/protocols.io.y2sfyee

Mrinalini Watsa

ABSTRACT

This protocol was used in the 2018 Genomics in the Jungle field course held at the Inkaterra Green Lab in the Madre de Dios Department of Peru. We were able to use it to take 112 amplicons and multiplex them using a 96 barcode kit on an Oxford Nanopore Technologies MinION sequencer. This protocol can be used to multiplex much larger numbers of samples onto a single flowcell if the amplicons are from differing taxonomic groups.

We multiplexed the following taxonomic groups:
1. Invertebrates
2. Mammals
3. Plants
4. Environmental DNA

We isolated amplicons using markers for:
1. rDNA (see Krehenwinkel, H., Pomerantz, A., Henderson, J.B., Kennedy, S.R., Lim, J.Y., Swamy, V., Shoobridge, J.D., Patel, N.H., Gillespie, R.G. and Prost, S., 2018. Nanopore sequencing of long ribosomal DNA amplicons enables portable and simple biodiversity assessments with high phylogenetic resolution across broad taxonomic scale. *bioRxiv*, p.358572.)
2. COI using a mammal cocktail as recommended in Kress, W.J. and Erickson, D.L., 2012. DNA barcodes: methods and protocols. In *DNA Barcodes* (pp. 3-8). Humana Press, Totowa, NJ.
3. rBCL and matK for plant samples

STEPS MATERIALS

| NAME | CATALOG # | VENDOR |
|---|---|---|
| GoTaq(R) G2 Hot Start Polymerase, Sample | M7402 | Promega |
| Q5 High-Fidelity 2X Master Mix - 500 rxns | M0492L | New England Biolabs |
| Agencourt Ampure XP | A63880 | Beckman Coulter |

BEFORE STARTING

We assume that this protocol begins with extracted DNA, and thus we do not detail DNA extraction protocols at this time.

## PCRMixes

1 1We used a master mix (See below) to set up PCR reactions



This is an open access protocol distributed under the terms of the **Creative Commons Attribution License** (https://creativecommons.org/licenses/by/4.0/), which permits unrestricted use, distribution, and reproduction in any medium, provided the original author and source are credited

GoTaq(R) G2 Hot Start Polymerase, Sample
by Promega
Catalog #: M7402

Q5 High-Fidelity 2X Master Mix - 500 rxns
by New England Biolabs
Catalog #: M0492L

**Butterfly rDNA (Total vol = 25 µl )**

1.25 µl each of the forward and reverse primer set

9.5 µl l water

3 µl **template** DNA

10 µl q5 MasterMix

**Mammal rDNA (Total vol = 13.5 µl )**

1 µl each of the forward and reverse primer set

2 µl l water

3 µl **template** DNA

6.5 µl q5 MasterMix

**Plant rDNA (Total vol = 13.5 µl )**

1 µl each of the forward and reverse primer set

2 µl l water

3 µl **template** DNA

6.5 µl q5 MasterMix

**Butterfly COI (Total vol = 25 µl )**

1.25 µl each of the forward and reverse primer set

7 µl l water

3 µl **template** DNA

12.5 µl q5 MasterMix

**Plant rBCL and matK (Total vol = 25 µl )**

1.25 µl each of the forward and reverse primer set

7 µl l water

3 µl **template** DNA

12.5 µl q5 MasterMix

**Ectoparasites, eDNA, dung beetles, snails COI (Total vol = 12.5 µl )**

2.5 µl Taq buffer



This is an open access protocol distributed under the terms of the **Creative Commons Attribution License** (https://creativecommons.org/licenses/by/4.0/), which permits unrestricted use, distribution, and reproduction in any medium, provided the original author and source are credited

1.25 µl MGCl2

0.0625 µl dNTPs (10mM)

0.125 µl each of the forward and reverse primer set

6.3775 µl l water

2 µl **template** DNA

0.06 µl **Hotstart GoTaq**

**Dung beetles,** ectoparasites, butterflies **(Total vol =** 12.5 µl **)**

2.5 µl Taq buffer

2 µl MGCl2

0.0625 µl dNTPs (10mM)

0.125 µl each of the forward and reverse primer set

5.63 µl l water

2 µl **template** DNA

0.06 µl **Hotstart GoTaq**

PCR conditions

2 rDNA PCR conditions

- **Initial denaturation for** 00:00:30 **at** 98 °C
- **Denaturation for** 00:00:10 **at** 98 °C
- **Annealing for** 00:00:30 **at** 68 °C
- **Extension for** 00:02:40 **at** 72 °C
- **Repeat cycles 35 times**
- **Final extention for** 00:02:00 **at** 72 °C

**COI (2 sets of conditions, but run one after the other since our program could not handle 2 sets at once.)**

- **Initial denaturation for** 00:02:00 **at** 94 °C
- **Denaturation for** 00:00:30 **at** 94 °C
- **Annealing for** 00:00:40 **at** 50 °C
- **Extension for** 00:01:00 **at** 72 °C
- **Repeat cycles 5 times**
- **Final extention for** 00:01:00 **at** 94 °C
- **Initial denaturation for** 00:00:01 **at** 94 °C
- **Denaturation for** 00:00:30 **at** 94 °C
- **Annealing for** 00:00:40 **at** 52 °C
- **Extension for** 00:01:00 **at** 72 °C
- **Repeat this set for 35 cycles**
- **Final extention for** 00:10:00 **at** 72 °C



This is an open access protocol distributed under the terms of the **Creative Commons Attribution License** (https://creativecommons.org/licenses/by/4.0/), which permits unrestricted use, distribution, and reproduction in any medium, provided the original author and source are credited

**Bioinformatics Pipelines/Practice Exercises:**

**Tools to Install**

| **Tool name** | **Link to source** | **Requirements** |
|---|---|---|
| Guppy | Available for download in your ONT account | Requires an account, which is only available once you purhcase a MinION. |
| Qcat | https://github.com/nanoporetech/qcat | Linux or MacOS |
| Nanofilt | https://github.com/wdecoster/nanofilt | None |
| Nanoplot | https://github.com/wdecoster/NanoPlot | Also available as a web service for small runs here |
| Canu | https://github.com/marbl/canu | None |
| Seqtk | https://github.com/lh3/seqtk | Requires zlib installed. |
| Minimap2 | https://github.com/lh3/minimap2 | Precompiled release available |
| Racon | https://github.com/isovic/racon | gcc 4.8+ or clang 3.4+<br>cmake 3.2+ |
| Cutadapt | https://github.com/marcelm/cutadapt | None |

**Basecall data using guppy**

~/ont-guppy-cpu/bin/guppy_basecaller -i ~/1.Fast5/Jungle1/20190723_2319_MN28056_FAK86661_99bcab5d/fast5_fail -s ~/2.Basecalled/trial1_fast5fail --flowcell FLO-MIN106 --kit SQK-LSK109 -t 8 --calib_detect

#Repeat for all files in pass and fail folders. Guppy automatically classifies reades with a quality score of 7 and below as failed, but we typically basecall all reads and then filter for quality after reviewing the quality of the whole run

**Combine all files into a single file.**

```
mv *.fastq ~/2a.combined_bc
```

**Demultiplex with qcat**

```
~/qcat -f /2a.combined_bc/joint.fastq -b /2b.demultiplexed
```

**Nanofilt**

```
for i in $(seq -w 1 96)
do

        nanofilt -l 250 /2b.demultiplexed/barcode${i}.fastq > /2c.Nanofilt/barcode${i}_filt.fastq

done
```

**Nanoplot for the whole run**

```
nanoplot --summary /2.Basecalled/trial2_fast5pass/sequencing_summary.txt --N50 -o /2d.Nanoplot
```

**Option 1: The following script uses canu/racon/minimap to take multiplexed amplicons sequenced with nanopore technology and create consensus sequences that have been error corrected and polished.**

```
#!/bin/bash
cd ~/data/
mkdir 3.canu;
mkdir 4.fastq_to_fasta;
mkdir 5.minimap1;
mkdir 6.racon1;
mkdir 7.minimap2;
mkdir 8.racon2;
mkdir 9.minimap3;
mkdir 10.racon3;
mkdir 11.cutadapt_top_output;
mkdir 12.cutadapt_untrimmed_top;
mkdir 13.cutadapt_bottom_output;
mkdir 14.cutadapt_untrimmed_bottom;
mkdir 15.joint_trimmed_reads;
mkdir 16.medaka;
```

```
for i in [list barcode numbers or range]
do

# 1) canu
	~/canu-1.8/Darwin-amd64/bin/canu -p barcode${i}_filt_canu -d ~/3.canu/barcode${i}_filt_canu genomeSize=2000 minReadLength=100 minOverLapLength=50 -nanopore-raw/2c.Nanofilt/barcode${i}_filt.fastq

# 2) fastq_to_fasta
	seqtk seq -A 1.joint_fastq/barcode${i}_filt.fastq > 4.fastq_to_fasta/barcode${i}.fasta;

# 3) minimap 1
	minimap2  3.canu/barcode${i}_filt_canu.contigs.fasta    4.fastq_to_fasta/barcode${i}.fasta > 5.minimap1/barcode${i}_map_1.paf;

# 4) racon 1
	racon  4.fastq_to_fasta/barcode${i}.fasta  5.minimap1/barcode${i}_map_1.paf 3.canu/barcode${i}_filt_canu.contigs.fasta >  6.racon1/barcode${i}_canu.racon1.fasta;

# 5) minimap 2
	minimap2  6.racon1/barcode${i}_canu.racon1.fasta   4.fastq_to_fasta/barcode${i}.fasta > 7.minimap2/barcode${i}_map_2.paf;

# 6) racon 2
	racon  4.fastq_to_fasta/barcode${i}.fasta  7.minimap2/barcode${i}_map_2.paf 6.racon1/barcode${i}_canu.racon1.fasta >  8.racon2/barcode${i}_canu.racon2.fasta;

# 7) minimap 3
	minimap2  8.racon2/barcode${i}_canu.racon2.fasta  4.fastq_to_fasta/barcode${i}.fasta > 9.minimap3/barcode${i}_map_3.paf;

# 8) racon 3
	racon 4.fastq_to_fasta/barcode${i}.fasta 9.minimap3/barcode${i}_map_3.paf 8.racon2/barcode${i}_canu.racon2.fasta > 10.racon3/barcode${i}_canu.racon3.fasta;

# 9) Trim top strand, shoot only trimmed into output file.

	cutadapt -g TTTCTGTTGGTGCTGATATTGC...GAAGATAGAGCGACAGGCAAGT 10.racon3/barcode${i}_canu.racon3.fasta -o 11.cutadapt_top_output/B${i}.toptrim.fasta --untrimmed-output 12.cutadapt_untrimmed_top/B${i}.untrimmed1.fasta > 11.cutadapt_top_output/B${i}.top.report.txt;
```

# 10) Trim bottom strand based on original racon output, this way a contig with both top and bottom chimeras will get split correctly into two trimmed contigs. Output contains only trimmed files.

cutadapt -g ACTTGCCTGTCGCTCTATCTTC...GCAATATCAGCACCAACAGAAA 10.racon3/barcode${i}_canu.racon3.fasta -o 13.cutadapt_bottom_output/B${i}.bottomtrim.fasta --untrimmed-output 14.cutadapt_untrimmed_bottom/B${i}.untrimmed2.fasta > 13.cutadapt_bottom_output/B${i}.bottom.report.txt;

# 11) To get a single contig file with all trimmed output, including any split chimeras, concatenate the above two trimmed outputs

cat 11.cutadapt_top_output/B${i}.toptrim.fasta 13.cutadapt_bottom_output/B${i}.bottomtrim.fasta > 15.joint_trimmed_reads/B${i}.joint.trimmed.contigs.fasta;

done

**Option 2:** Alternatively, medaka (ONT; https://github.com/nanoporetech/medaka) can be used to replace #5 to #8. If so, use 16.medaka/barcode${i}_canu.medaka.fasta instead of 10.racon3/barcode${i}_canu.racon3.fasta in #9.

medaka_consensus-i 1.joint_fastq/barcode${i}_filt.fastq  - d 6.racon1/barcode${i}_canu.racon1.fasta -o 16.medaka/barcode${i}_canu.medaka.fasta

**Suggested Readings:**

| **Resource** | **Reference** |
| --- | --- |
| For a summary of DNA barcoding processes, including tips on making extraction buffers in house | *Kress WJ, Erickson DL*. DNA barcodes: methods and protocols. Methods Mol Biol. 2012;858: 3–8 |
| The paper describing the outlined pipeline. | *Pomerantz, A., Peñafiel, N., Arteaga, A., Bustamante, L., Pichardo, F., Coloma, L.A., Barrio-Amorós, C.L., Salazar-Valenzuela, D. and Prost, S.,* 2018. Real-time DNA barcoding in a rainforest using nanopore sequencing: opportunities for rapid biodiversity assessments and local capacity building. GigaScience, 7(4), p.giy033. |
| The seminal paper on DNA barcoding that began the whole barcode of life movement | *Hebert, P., Cywinska, A., Ball, S., deWaard, J.* (2003). Biological identifications through DNA barcodes Proceedings of the Royal Society of London. Series B: Biological Sciences 270(1512), 313-321. https://dx.doi.org/10.1098/rspb.2002.2218 |
| Details on barcoding plants, which need subsequent multi-gene phylogenetics | *Kress, W., Erickson, D., Jones, F., Swenson, N., Perez, R., Sanjur, O., Bermingham, E.* (2009). Plant DNA barcodes and a community phylogeny of a tropical forest dynamics plot in Panama Proceedings of the National Academy of Sciences of the United States of America 106(44), pnas.0909820106. https://dx.doi.org/10.1073/pnas.0909820106 |
| A field-specific workflow for tracking species biodiversity using the MinION. | *Maestri, S., Cosentino, E., Paterno, M., Freitag, H., Garces, J., Marcolungo, L., Alfano, M., Njunjić, I., Schilthuizen, M., Slik, F., Menegon, M., Rossato, M., Delledonne, M.* (2019). A Rapid and Accurate MinION-Based Workflow for Tracking Species Biodiversity in the Field Genes 10(6), 468. https://dx.doi.org/10.3390/genes10060468 |
| Use of the MinION in remote field stations in Madagascar for local capacity building. | *Blanco, M., Greene, L., Williams, R., Andrianandrasana, L., Yoder, A., Larsen, P. (2019).* ***Next-generation in situ conservation and educational outreach in Madagascar using a mobile genetics lab*** *bioRxiv https://dx.doi.org/10.1101/650614* |

## Metabarcoding

**Goal:** The amplification of DNA barcodes using universal primers to detect many taxa within a bulk community or pooled taxon sample.

**Recommended Samples**: These samples can be from various sources: a) pooled insects from a Malaise trap, b) fecal sample from any source, c) any other bulk sample. For microbiome analyses, 16S primers and pipelines are well documented in the literature [2].

**Ingredients**:A full ingredient list is linked to the protocol below (also available on protocols.io.dx.doi.org/10.17504/protocols.io.y2pfydn) but in general terms includes:

- A DNA extraction kit
- Or in-house extraction buffers [see 1] + spin columns
- Primers
- PCR master mix or taq+dNTPs
- Barcoding kit or custom barcodes
- dA-tailing and end-repair kit (New England Biolabs)
- Library prep kit (ONT)

**Laboratory Protocol:**

Below, find a protocol for 16S metabarcoding of fecal samples, with online versions available here, and here (for 18S).

# 16S Metagenomics in a Field Setting V.3

Mar 11, 2019

Gideon Erkenswick[1,2], Stefan Prost[3,4], Mrinalini Watsa[1,5,2], Aaron Pomerantz[6,7]

[1]University of Missouri - Saint Louis, [2]Field Projects International, [3]LOEWE-Center for Translational Biodiversity Genomics, Senckenberg Museum, 60325 Frankfurt, Germany, [4]South African National Biodiversity Institute, National Zoological Garden, Pretoria 0184, South Africa, [5]Washington University in Saint Louis, [6]University of California, Berkeley, CA, USA, Department of Integrative Biology, [7]Marine Biological Laboratory, Woods Hole, MA - USA

1 Works for me dx.doi.org/10.17504/protocols.io.y2pfydn

Mrinalini Watsa

ABSTRACT

This protocol is was used to conduct DNA 16S metagenomics on FPI's Genomics in the Jungle - 2018 field course at the Green Lab, located and Inkaterra Guides Field Station, Madre de Dios, Peru.

GUIDELINES

This protocol starts from already extracted DNA

STEPS MATERIALS

| NAME | CATALOG # | VENDOR |
|---|---|---|
| Agencourt Ampure XP | A63880 | Beckman Coulter |

## Amplification

1 **Remove samples and the following reagents and let thaw, once thawed keep on ice block**

- 10mM DNTPs
- 25 mMgCl
- 5x Go Taq Buffer
- Forward primer 16S-27F (10uM)
- Reverse primer 16S-1429R (10uM)
- GoTaq Hotstart Polymerase 5u/ul

**Make PCR cocktail for # of samples * 1.1** (10% extra). Don't forget to include 1 PCR negative control for each separate PCR

**Run PCR according to the following cycle conditions:**

- Initial denaturation **95 °C** for 120s
- 25 cycles of **95 °C** for 30s, **51 °C** for 30s, **72 °C** for 30s
- Final extension **72 °C** for 420s

Upon completion remove, label, and store at **4 °C** , or take directly to electrophoresis



This is an open access protocol distributed under the terms of the **Creative Commons Attribution License** (https://creativecommons.org/licenses/by/4.0/), which permits unrestricted use, distribution, and reproduction in any medium, provided the original author and source are credited

Electrophoresis

2 **Equipment**

- BlueGel system
- MiniOne system

**Create .8 - 1.0% agarose 1 gel with 13 combs**

- Measure 1 g of agarose
- Mix agraose with 100 mL of 1xTBE
- Microwave the mixture until agarose is completely dissolved (1-3 min)
- Pour the agarose gel into the tray with the comb in place.
- Allow the agarose gel to harden (20-30 min)

**Insert the agarose gel into electrophoresis equipment and add 1xTBE buffer until the agarose gel is submerged**

**Spot check with 2 µl of each sample**

**Mix 1 µl of loading dye to 2 µl of each sample and load the gel.** (If Green Taq buffer with built in loading dye was used, skip this step).

**Load 5 µl of100kp ladder into the agarose gel.**

**Turn on the electrode and let the DNA run until the band is identifiable (**

Barcoding PCR

3
- A barcoding PCR was run to attach barcodes from the 96-barcode kit for the MinION to each sample
- We did not use special PCR mastermix at this stage, using instead a mix similar to that of the PCRs above
- We used 1 µl of each barcode primer and 2 µl of every postive PCR amplicon in a total volume of 25 uL

We ran the PCR at the following conditions:

- Initil denaturation of hotstart taq at 95 °C for 00:02:00
- Denaturation at 95 °C for 00:00:30
- Annealing at 62 °C for 00:00:30
- Extension at 72 °C for 00:00:45
- Total number of cycles - 18
- Final Extension at 72 °C for 00:05:00



This is an open access protocol distributed under the terms of the **Creative Commons Attribution License** (https://creativecommons.org/licenses/by/4.0/), which permits unrestricted use, distribution, and reproduction in any medium, provided the original author and source are credited

**Bioinformatics Pipelines/Practice Exercises:**

**Tools to Install**

| **Tool name** | **Link to source** | **Requirements** |
|---|---|---|
| Guppy | In ONT accounts | |
| Qcat | https://github.com/nanoporetech/qcat | Linux or MacOS |
| Nanofilt | https://github.com/wdecoster/nanofilt | None |
| Nanoplot | https://github.com/wdecoster/NanoPlot | Also available as a web service for small runs here |
| WIMP: What's in my Pot | See details here | An ONT account, available only upon purchase of a MinION or higher. |

**Basecall data using guppy**

~/ont-guppy-cpu/bin/guppy_basecaller -i ~/1.Fast5/Jungle1/20190723_2319_MN28056_FAK86661_99bcab5d/fast5_fail -s ~/2.Basecalled/trial1_fast5fail --flowcell FLO-MIN106 --kit SQK-LSK109 -t 8 --calib_detect

#Repeat for all files in pass and fail folders. Guppy automatically classifies reades with a quality score of 7 and below as failed, but we typically basecall all reads and then filter for quality after reviewing the quality of the whole run

**Combine all files into a single file.**

mv *.fastq ~/2a.combined_bc

**Demultiplex with qcat (if multiple samples were pooled on the same run)**

~/qcat -f /2a.combined_bc/joint.fastq -b /2b.demultiplexed

**Nanofilt**

for i in $(seq -w 1 96)
do

```
nanofilt -l 250 /2b.demultiplexed/barcode${i}.fastq > /2c.Nanofilt/barcode${i}_filt.fastq
done
```

**Nanoplot for the whole run**

```
nanoplot --summary /2.Basecalled/trial2_fast5pass/sequencing_summary.txt --N50 -o /2d.Nanoplot
```

**WIMP-based Analysis**

Upload the respective barcode fastq files to the ONT server using EPI2ME. See https://nanoporetech.com/nanopore-sequencing-data-analysis

**BLAST**

Alternatively, reads can be blasted to NCBI online at:

https://blast.ncbi.nlm.nih.gov/Blast.cgi

**Additional Suggested Readings:**

| **Resource** | **Reference** |
|---|---|
| Field-based analysis of microbial communities on an expedition. | *Gowers, G., Vince, O., Charles, J., Klarenberg, I., Ellis, T., Edwards, A.* (2019). Entirely Off-Grid and Solar-Powered DNA Sequencing of Microbial Communities during an Ice Cap Traverse Expedition. Genes 10(11), 902. https://dx.doi.org/10.3390/genes10110902 |

## eDNA: Environmental DNA Monitoring

**Goal:** Metabarcoding for environmental samples to pick up trace DNA left by organisms living in the environment.

**Recommended Samples**: These samples can be from various sources: a) water samples from a lake or stream, b) soil samples, c) sediments from water bodies, d) leaf litter samples. Target strategies can take two forms: first, the detection of a single or few target species, such as in the case of invasive species monitoring or rare-species detection or second, the screening of an entire community using generalised markers.

**Ingredients**: A full ingredient list in general terms includes:

- A filtration system for straining biological materials out of an environmental sample onto a filter
- A DNA extraction kit
- Or in-house extraction buffers [see 1] + spin columns
- Primers
- PCR master mix or taq+dNTPs
- Barcoding kit or custom barcodes
- dA-tailing and end-repair kit (New England Biolabs)
- Library prep kit (ONT)

**Laboratory Protocol:**

In order to plan a successful environmental DNA experiment, one must consider several factors. These include the implementation of controls and other checks to ensure that the number of reads can be correlated with confidence to the prevalence of a species in a community. As such, experiments vary in both scope and complexity, depending on desired outcomes. The basic laboratory protocol follows those listed above for metabarcoding using broad markers.

**Bioinformatics Pipelines/Practice Exercises:**

**Tools to Install**

| **Tool name** | **Link to source** | **Requirements** |
|---|---|---|
| Guppy | In ONT accounts | |
| Qcat | https://github.com/nanoporetech/qcat | Linux or MacOS |
| Nanofilt | https://github.com/wdecoster/nanofilt | None |

| | | |
|---|---|---|
| Nanoplot | https://github.com/wdecoster/NanoPlot | Also available as a web service for small runs here |
| WIMP: What's in my Pot | See details here | An ONT account, available only upon purchase of a MinION or higher. |

**Basecall data using guppy**

~/ont-guppy-cpu/bin/guppy_basecaller -i ~/1.Fast5/Jungle1/20190723_2319_MN28056_FAK86661_99bcab5d/fast5_fail -s ~/2.Basecalled/trial1_fast5fail --flowcell FLO-MIN106 --kit SQK-LSK109 -t 8 --calib_detect

#Repeat for all files in pass and fail folders. Guppy automatically classifies reades with a quality score of 7 and below as failed, but we typically basecall all reads and then filter for quality after reviewing the quality of the whole run

**Combine all files into a single file.**

mv *.fastq ~/2a.combined_bc

**Demultiplex with qcat (if multiple samples were pooled on the same run)**

~/qcat -f /2a.combined_bc/joint.fastq -b /2b.demultiplexed

**Nanofilt**

```
for i in $(seq -w 1 96)
do

        nanofilt -l 250 /2b.demultiplexed/barcode${i}.fastq > /2c.Nanofilt/barcode${i}_filt.fastq

done
```

**Nanoplot for the whole run**

nanoplot --summary /2.Basecalled/trial2_fast5pass/sequencing_summary.txt --N50 -o /2d.Nanoplot

**WIMP-based Analysis**

Upload the respective barcode fastq files to the ONT server using EPI2ME. See https://nanoporetech.com/nanopore-sequencing-data-analysis

**BLAST**

Alternatively, reads can be blasted to NCBI online at:

https://blast.ncbi.nlm.nih.gov/Blast.cgi

**Suggested Readings:**

| **Resource** | **Reference** |
|---|---|
| Seminal paper on environmental DNA detection from soil samples | *Handelsman, J., Rondon, M., Brady, S., Clardy, J., Goodman, R.* (1998). Molecular biological access to the chemistry of unknown soil microbes: a new frontier for natural products Chemistry & Biology 5(10), R245-R249. https://dx.doi.org/10.1016/s1074-5521(98)90108-9 |
| Dealing with error rates in eDNA sampling | *Ficetola, G., Pansu, J., Bonin, A., Coissac, E., Giguet-Covex, C., Barba, M., Gielly, L., Lopes, C., Boyer, F., Pompanon, F., Rayé, G., Taberlet, P.* (2015). Replication levels, false presences and the estimation of the presence/absence from eDNA metabarcoding data Molecular Ecology Resources 15(3), 543-556. https://dx.doi.org/10.1111/1755-0998.12338 |

**References:**


1. Kress WJ, Erickson DL. DNA barcodes: methods and protocols. Methods Mol Biol. 2012;858: 3–8.

2. Klindworth A, Pruesse E, Schweer T, Peplies J, Quast C, Horn M, et al. Evaluation of general 16S ribosomal RNA gene PCR primers for classical and next-generation sequencing-based diversity studies. Nucleic Acids Res. 2013;41: e1.
